# Linker-Free Synthesis of Antimicrobial Peptides Using a Novel Cleavage Reagent: Characterisation of the Molecular and Ionic Composition by nanoESI-HR MS

**DOI:** 10.3390/pharmaceutics15041310

**Published:** 2023-04-21

**Authors:** Roser Segovia, Mireia Díaz-Lobo, Yolanda Cajal, Marta Vilaseca, Francesc Rabanal

**Affiliations:** 1 Section of Organic Chemistry, Department of Inorganic and Organic Chemistry, Faculty of Chemistry, Universitat de Barcelona, 08028 Barcelona, Spain; rosersegovia@gmail.com; 2 Institute for Research in Biomedicine (IRB Barcelona), BIST (The Barcelona Institute of Science and Technology), Baldiri Reixac 10, 08028 Barcelona, Spain; mireia.diaz@irbbarcelona.org (M.D.-L.); marta.vilaseca@irbbarcelona.org (M.V.); 3 Department of Pharmacy, Pharmaceutical Technology and Physical Chemistry, Faculty of Pharmacy and Food Sciences, Universitat de Barcelona, 08028 Barcelona, Spain; ycajal@ub.edu; 4 Institute of Nanoscience and Nanotechnology (IN2UB), Universitat de Barcelona, 08028 Barcelona, Spain

**Keywords:** peptide, synthesis, Fmoc, antimicrobial, cleavage, ionic, characterisation

## Abstract

The efficient preparation of novel bioactive peptide drugs requires the availability of reliable and accessible chemical methodologies together with suitable analytical techniques for the full characterisation of the synthesised compounds. Herein, we describe a novel acidolytic method with application to the synthesis of cyclic and linear peptides involving benzyl-type protection. The process consists of the in situ generation of anhydrous hydrogen bromide and a trialkylsilyl bromide that acts as protic and Lewis acid reagents. This method proved to be useful to effectively remove benzyl-type protecting groups and cleave Fmoc/^t^Bu assembled peptides directly attached to 4-methylbenzhydrylamine (MBHA) resins with no need for using mild trifluoroacetic acid labile linkers. The novel methodology was successful in synthesising three antimicrobial peptides, including the cyclic compound polymyxin B3, dusquetide, and RR4 heptapeptide. Furthermore, electrospray mass spectrometry (ESI-MS) is successfully used for the full characterisation of both the molecular and ionic composition of the synthetic peptides.

## 1. Introduction

The synthesis of peptides is in constant need of new advances for efficient production both at the laboratory and industrial scales. Nowadays, peptide drugs are routinely prepared at kilogram-scale by specialised manufacturing companies. Notwithstanding that, it is evident that small optimisation steps in peptide chemistry may imply substantial improvements in the large-scale process. A key step in peptide synthesis is the final cleavage, during which multiple protecting groups are removed. In the case of the solid-phase strategy, the peptide is also detached from the resin.

Currently, the solid-phase synthesis of peptides is generally performed following the Fmoc/^t^Bu chemistry that has mostly displaced the original Boc/Bn strategy described by Merrifield [1]. The reason lies in the use of milder acids such as trifluoroacetic acid (TFA) instead of the harsh anhydrous HF conditions needed to cleave benzyl-type protecting groups and linkers, which facilitate the cleavage step [2]. However, these milder cleavage conditions generally require the use of finely tuned linkers to adjust the lability of the peptide-resin bond to TFA. For instance, the Rink linker needs to be compared to the benzhydrylamine resin in the Boc/Bn chemistry. Although milder cleavage conditions may require fine-tuned linkers, we believe that an acidolytic method that can easily cleave benzyl-type protecting groups and peptides bound to derivatized resins (such as MBHA) would still be useful.

In 1952, Ben-Ishai and Berder were engaged in finding a technique for eliminating benzyl-protecting groups [3]. They demonstrated that benzyl carbamates and carbobenzoxy-α-amino acids are cleaved at room temperature by hydrogen bromide in acetic acid (HBr/AcOH) to the corresponding ammonium hydrobromides, benzyl bromide, and carbon dioxide. This deprotecting procedure was then applied to the solid-phase peptide synthesis by Merrifield in 1963 [4]. However, this method did not gain wide acceptance in peptide synthesis because of the presence of AcOH-generated acetylated by-products. Therefore, the anhydrous hydrogen fluoride (HF) methodology reported by Sakakibara et al., in 1967 [5] was found to be more useful for small-scale synthesis. The method involved an acidic cleavage of the peptidyl resin, which was placed in an HF-reaction cylinder and treated with anhydrous HF and anisole. After one hour at 0 °C, the excess HF was removed, and the released peptide was extracted with water.

Nowadays, the procedure described by Tam et al., in 1983 [6], consisting of a two-stage procedure named the “low-high HF deprotection procedure,” is the most widely applied for the cleavage of Boc/Bn-protected peptides. It incorporates an initial cleavage of most of the side chain protecting groups under treatment with HF, dimethylsulfide, and *p*-cresol (25:65:10, *v*/*v*/*v*), followed by a second standard HF cleavage step to remove the more resistant protecting groups. Nevertheless, this methodology still presents several drawbacks, such as the need to handle the extremely toxic and volatile HF and its corrosive action on glass. This requirement demands the use of special and costly Teflon^®^ labware and HF-resistant fume hoods.

Other strong acids, such as trifluoromethanesulfonic acid (TFSMA) can be used as an alternative to HF. In 1974 Yajima et al. [7] reported the use of TFMSA in TFA, which was later refined by Tam et al., in 1986 [8] They proposed the system TFMSA-TFA-Me_2_S. This cleavage procedure is widely applied in peptide synthesis since it does not require special equipment and can be adapted to large-scale processes However, it also presents some disadvantages, such as lower yields compared to the HF procedure and the fact that TFMSA is not volatile.

In addition, other acidic treatments based on silicon compounds have been proven to be useful methods for the deprotection and cleavage of peptides. Yajima et al. [9] and Hughes et al. [10] substituted the use of TFMSA with TMSOTf or TMSBr, which act as strong Lewis acids when combined with thioanisole and TFAThese methods have been shown to produce comparable results to those obtained by the TFMSA or HF treatments.

Catalytic hydrogenation also gained wide acceptance as an easy method to cleave hydrogenolysable while protecting groups. This technique offers some advantages in peptide synthesis since it potentially reduces the risk of damaging the peptide due to the absence of strong acidic conditions, which can be harmful to highly sensitive peptides [11,12]. However, it presents considerable hazards because of the use of molecular hydrogen, a gas of low molecular weight, high diffusibility, and ignitability that becomes even more fire-sensitive in the presence of the palladium catalysts (i.e., Pd/C). As an alternative, the catalytic transfer hydrogenation method (CTH) was introduced by Jackson and Johnstone [13] in 1976. In this method, a donor generates hydrogen gas in the presence of Pd/C. In that regard, ammonium formate, sodium formate, cyclohexadiene, cyclohexene, or sodium hypophosphite are commonly used as hydrogen donors [14]. This method is extremely rapid, economical, safe, and therefore more convenient than the standard hydrogenation reaction when applied to peptides synthesised in solution. However, applying this method to solid-phase peptide synthesis presents a challenge, as cleavage of the peptide from the resin involves poor solid–solid interactions between the support and the catalyst, which usually results in low cleavage yields.

In this paper, we present a novel acidolysis reagent, which consists of anhydrous HBr and a Lewis silyl acid prepared by the in situ reaction of bromine and silane in TFA. We demonstrate the usefulness and application of this method in the chemical synthesis of both cyclic and linear antimicrobial peptides. Examples of peptides include the cyclic lipopeptide polymyxin B_3_, linear dusquetide, and RR4 heptapeptide fragment, which contains both tryptophan and arginine in the sequence. This combination is known to be challenging during the cleavage step, making it an important application of the method. Polymyxin B is an old antimicrobial peptide that has recently been reintroduced into clinical practise due to the alarming increase in bacterial resistance to commonly used antibiotics. Currently, it is being extensively studied to develop new analogues with improved therapeutic windows [15,16,17,18,19,20,21]. Dusquetide is a synthetic 5-amino acid compound derived from the antimicrobial peptide indolicin. It is currently in Phase III clinical trials for the treatment of oral mucositis in patients undergoing concomitant chemoradiation therapy for head and neck cancer [22,23]. Finally, RR4 is a rationally designed peptide composed of 14 amino acid residues that present significant activity against multi-drug resistant *Pseudomonas aeruginosa* and *Acinetobacter baumannii* [24].

## 2. Materials and Methods

### 2.1. Chemicals

4-Methylbenzhydrylamine hydrochloride resin (MBHA), 2-Chlorotrityl chloride resin (2-CTC), *N*-fluorenylmethoxycarbonyl (Fmoc)-protected amino acids, trifluoroacetic acid (TFA), and *N*-hydroxybenzotriazole (HOBt) were purchased from Fluorochem (Hadfield, UK) and Iris Biotech GmbH (Marktredwitz, Germany). Octanoic acid, acid, and *N*,*N*’-diisopropylcarbodiimide (DIC) from Thermo Fisher Scientific (Waltham, MA, USA) and Fluka (Buchs, Switzerland). Acetonitrile HPLC gradient grade was purchased from Labkem (Barcelona, Spain). Polymyxin B sulphate salts were purchased from Sigma Aldrich (St. Louis, MO, USA). All chemicals were of the highest available purity. Water was doubly distilled and deionized (Milli-Q system, Millipore Corp., Burlington, MA, USA).

### 2.2. Peptide Synthesis and Purification

Manual solid-phase peptide synthesis was performed following standard Fmoc/Bn/^t^Bu or Fmoc/^t^Bu protection strategies using a 3-fold molar excess of amino acid/DIC/HOBt activation on the 2-CTC resin (f = 1.67 mmol/g of resin) or MBHA resin (f = 0.69 mmol/g of resin) in a polypropylene syringe fitted with a polyethylene disc. All coupling reactions were performed in the minimum possible amount of DMF for one hour with occasional manual stirring at room temperature, and then the resin was drained and washed with DMF and DCM (5 × 1 min). All coupling reactions proceeded to greater than 99% completion as assessed by Kaiser tests. After each coupling step, the N-terminal Fmoc group was removed with 20% piperidine in DMF (1 × 1 min, 2 × 10 min).

In the case of PxB_3_, on peptide completion, the partially protected peptide was obtained after cleavage with 5 mL of DCM/TFA/TES/H_2_O (55:40:3:2, *v*/*v*/*v*/*v*) for 30 min. The resin was then removed by filtration and washed twice with 2 mL of the cleavage mixture. The combined filtrates were transferred to 50 mL microcentrifuge tubes, and the volume was reduced by evaporation using a stream of nitrogen. Thereafter, the peptide was precipitated, washed three times with cold water, and lyophilized to yield 339 mg of white powder (ŋ_cleavage_ = 82%). The partially protected PxB_3_ was cyclized using HATU, HOAt, and DIPEA in DMF. The cyclization was left to react for 2–3 h, and it was monitored by TLC, which was stained with ninhydrin. The cyclized protected peptide was then precipitated with a 10-fold excess of cold water, isolated by centrifugation, and lyophilized. The crude peptide yielded 257 mg (ŋ_cyclization_ = 81%). To further treat the peptide, a 100 mL acidic mixture composed of HBr, Et_3_SiBr, TFA, and TES, was used. The mixture was prepared from TFA/TES/Br_2_ (82.5:15:2.5, *v*/*v*/*v*) at 0 °C. Careful addition was required, as heating and bubbling occurred. The peptide was treated with the acidic mixture for 45 min. After the acidic treatment, the peptide solution was evaporated under a gentle stream of nitrogen gas to a reduced volume. The resulting crude product was then precipitated and washed three times with Et2O, yielding a total amount of 184 mg (ŋ_acidolysis_ = 92%) of an orange/off-white solid. To obtain the pure peptide, semi-preparative HPLC was performed, resulting in 69 mg of white pure peptide (ŋ_purification_ = 34%). The overall yield for the process was 21%.

On the other hand, dusquetide and RR4 peptidyl resins were treated with DCM/TFA (1:1, *v*/*v*) for 1 min. Afterward, the peptidyl resins were cleaved with the HBr solution previously prepared from TFA/TES/Br_2_ (82.5:15:2.5, *v*/*v*/*v*) for 90 min. TIS was used in the synthesis of RR4. The peptide solution was evaporated under a gentle stream of nitrogen gas to a reduced volume, and the crude product was precipitated and washed three times with anhydrous Et_2_O. The peptides were obtained with an orange/off-white coloration that disappeared after three lyophilization cycles with 5 mL H_2_O/ACN (1:1, *v*/*v*). No further purification was performed.

The homogeneity of peptide crudes was assessed by analytical HPLC on Nucleosil C18 reverse-phase columns (4 mm × 250 mm, 5 μm particle diameter, and 120 Å porous size). Elution was carried out at 1 mL·min^−1^ flow with mixtures of H_2_O/0.045% TFA and acetonitrile/0.036% TFA and UV detection at 220 nm. PxB_3_ was purified by preparative HPLC on a Waters Delta Prep 3000 system using a Phenomenex C18 column (250 mm × 10 mm, 5 μm) eluted with H_2_O/ACN/0.1% TFA gradient mixtures and UV detection at 220 nm.

### 2.3. Peptide Counterion Exchange

Pure lipopeptides obtained as trifluoroacetate salts were subjected to three cycles of dilution and lyophilization with HCl (8 mM). Solutions of 1 mg·mL^−1^ were prepared, thoroughly vortexed, and immediately frozen and lyophilized. The completion of the counterion exchange was assessed by quantitative ^19^F-NMR spectroscopy.

The sulphate counterions of commercial polymyxin B were also exchanged through the acquisition of the free-base peptide. Polymyxin B was neutralised using equimolar amounts of KOH (0.1 M). The resulting peptide, completely insoluble in neutral aqueous conditions, was isolated by centrifugation (6000 rpm, 10 min, 4 °C) and washed three times with cold water. The obtained free-base polymyxin was treated with equimolar amounts of HCl (8 mM) until its complete dissolution and finally obtained as hydrochloride salt after lyophilisation.

### 2.4. CHNS Elemental Analysis

CHNS elemental analysis was carried out using an elemental organic analyser Thermo EA Flash 2000 (Thermo Scientific, Milan, Italy), working in standard conditions recommended by the supplier of the instrument (helium flow of 140 mL/min, a combustion furnace at 950 °C, and a chromatographic column oven at 65 °C). The samples (1.000 mg) were analysed in triplicate.

### 2.5. Chloride Determination by Titration

The chloride content of the peptides was analysed directly using a Mettler Toledo DL-50 Titrator (Mettler Toledo GmbH, Schwerzenbach, Switzerland) equipped with a MT Silver-Ring Electrode (ref. DM-141-SC) and a 0.01 M aqueous solution of silver nitrate. The samples were analysed in triplicate. Samples (4.000 mg) were weighted in a microbalance (MT MX5; Mettler Toledo) and dissolved in nitric acid (0.06 M; 30 mL).

### 2.6. Nano-Electrospray Ionisation High-Resolution Mass Spectrometry

Peptide samples were reconstituted with 100 μL of H_2_O and diluted by 1/3 in ACN/H_2_O for MS analysis. An Orbitrap Fusion Lumos™ Tribrid (Thermo Scientific, Waltham, MA, USA) mass spectrometer was used with an Advion Triversa Nanomate (Advion BioSciences, Ithaca, NY, USA) nanoelectrospray source working in negative polarity mode at 120 k resolution. The ion spray voltage was set to −1.7 kV, the ion transfer tube was kept at 275 °C, and the source pressure was set at 2.6 Torr. We used a 60% RF lens and acquired data over the 150–2000 *m*/*z* range at orbitrap resolution (120 k). Xcalibur software vs. 4.2.28.14 was used for data acquisition and processing. We used the integrated tool in Xcalibur software for elemental composition search of the *m*/*z* ions detected and for isotope pattern comparison with theoretical spectra. The detected *m*/*z* for all compounds and their corresponding molecular formulae and mass accuracy are detailed in Table S1 in the Supporting Information.

### 2.7. Electrospray Ionisation Mass Spectrometry

Peptide samples were reconstituted with 1 mL of ACN/H_2_O for MS analysis. A Waters instrument was employed, comprising a Waters 2996 photodiode array detector, a Waters ESI-MS Micromass ZQ 4000 spectrometer, and a Masslynx v4.1 system controller. In this case, we used a standard electrospray source. The electrospray needle voltage was set to −2 kV, and the heated capillary was kept at 300 °C. Detected *m*/*z* for all compounds and corresponding molecular formulae and mass accuracy are detailed in Tables S2 and S3 in the Supporting Information.

### 2.8. Antibacterial Susceptibility Testing

The antimicrobial activity of the synthesised peptides was determined in *Pseudomonas aeruginosa* ATCC 27853. Sterile microtiter plates (96 wells of 100 μL) were filled with 50 μL of Muller-Hinton broth (MHB) culture medium. Serial 2-fold dilutions of the peptides were arranged in rows ranging from 32 to 0.06 μg·mL^−1^. The last two columns of the plate were used as positive controls (no peptide) and negative controls (no peptide and not inoculated with bacteria), respectively. The bacterial suspensions prepared earlier with an OD_540_ of 0.2 were diluted 100-fold, and 50 μL of the resulting suspension was added to each well (excluding the final column). This resulted in a final concentration of approximately 10^6^ CFU·mL^−1^. The plates were incubated at 37 °C for 18–20 h. Bacterial growth was determined visually based on turbidity, and the MIC was considered to be the lowest peptide concentration inhibiting bacterial growth. Each determination was carried out in triplicate. To be considered acceptable, the three MIC results have to differ in only one well, and the result is always given as the higher of the three.

## 3. Results

### 3.1. Novel Cleavage Reagent

**Figure 1:**
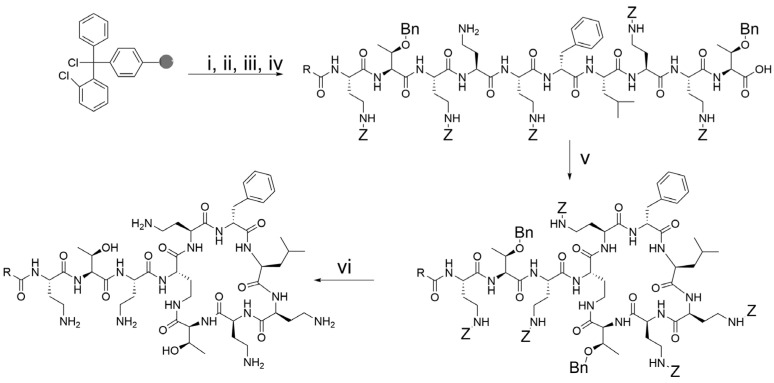
Scheme of the synthesis of polymyxin B_3_ (*n*-octyl-Dab-Thr-Dab-*cyclo*[Dab-Dab-DPhe-Leu-Dab-Dab-Thr]. (i) 1.2 eq of Fmoc-Thr(Bn)-OH and 4 eq of DIPEA in CH_2_Cl_2_ at room temperature, overnight; (ii) 20% of piperidine in DMF (1 × 1 min, 2 × 10 min); (iii) repetitive cycles of coupling and deprotection using a 3-fold molar excess of protected Fmoc-AA-OH (or octanoic acid at the end) and DIC/HOBt; (iv) CH_2_Cl_2_/TFA/TES/H_2_O (55:40:3:2) for 30 min at room temperature (ŋ_cleavage_ = 82%); (v) HATU/HOAt/DIPEA (2:2:4 eq) for 2–4 h, monitored by TLC stained with ninhydrin (ŋ_cyclization_ = 81%); and (vi) HBr/Et_3_Si-Br/TES/TFA, in situ prepared with TFA/TES/Br_2_ (82.5:15:2.5) for 45 min at room temperature (ŋ_acidolysis_ = 92%). Final yield: 21%, purity > 95%. R = C_7_H_15_ (corresponding to octanoyl).

The first steps towards the development of the novel acidolysis reagent for the deprotection of benzyl-type protecting groups, including benzyl ethers and urethanes such as the benzyloxycarbonyl group, also known as Z, were undertaken by testing different acidic mixtures based on anhydrous HBr. As the peptide model, we used the antimicrobial cyclic peptide polymyxin B_3_, which was synthesised following a Fmoc/Bn/^t^Bu protection strategy on a CTC resin as described in Figure 1. The chemical strategy consisted of using the different acid lability of Boc and the trityl resin to release a partially benzyl-protected linear precursor to direct the tail-to-side-chain macrolactam cyclization (Dab^3^-Thr^10^).

The final HBr acidolysis treatment was initially conducted on a small scale. Briefly, the cyclic-protected peptide (5 mg) was dissolved in 2 mL of the acidolysis reagent at different conditions as indicated in Table 1. The crude was obtained by precipitation in anhydrous cold diethyl ether and analysed by HPLC and ESI-MS spectrometry.

**Figure 2:**
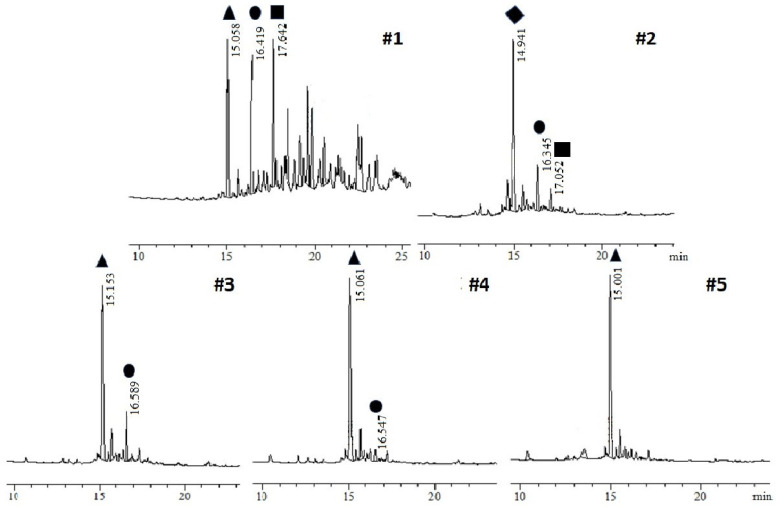
PxB_3_ crude HPLC chromatograms obtained by elution using a linear gradient from 5% to 95% of B over 30 min; eluants, A: 0.045% TFA in H_2_O and B: 0.036% TFA in acetonitrile. Crudes were obtained after treatment of #1, #2, #3, #4, and #5 (see Table 1 for detailed reaction conditions). ▲ = PxB_3_, C_55_H_96_N_16_O_13_, exact mass: 1188.73, found 1190.11 [M + H]^+^; ● = PxB_3_ + Bn, C_62_H_102_N_16_O_13_, exact mass: 1278.78, found 1279.72 [M + H]^+^; ■ = PxB_3_ + 2Bn, C_69_H_108_N_16_O_13_, exact mass: 1368.82, found 1370.10 [M + H]^+^; ◆ = PxB_3_ − 2H_2_O, C_55_H_92_N_16_O_11_, exact mass: 1152.71, found 1153.86 [M + H]^+^.

The first acidolysis test was performed with a reagent composed of HBr solution with 33 wt.% in AcOH, TMSCl, and TES [25] (entry #1 Table 1). After 2 h of treatment at room temperature, only a 6% yield of the expected cyclic peptide was obtained. The conditions used in the reaction, which contained 1.8 wt% of HBr along with 25.8 *v*/*v*% of TMSCl and 64.2 *v*/*v*% of TFA, appear to not be acidic enough to fully deprotect polymyxin B_3_. The crude yielded a highly complex mixture of partially deprotected peptides and other minor by-products (Figure 2, entry #1).

**Scheme 1:**
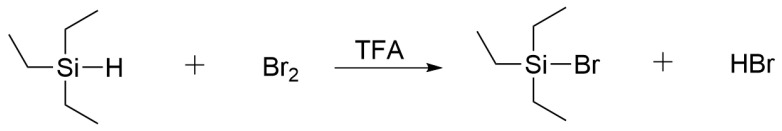
In situ hydrogen bromide generation by the reaction of triethylsilane with bromine.

To obtain stronger acidic mixtures and avoid the use of acetic acid, the in situ generation of HBr in TFA was explored. The generation of hydrogen halides by the reaction of halogens with trialkyl silanes was first described by Deans et al., in 1954 [26]. We tested the reaction of triethylsilane (TES) and bromine in TFA to prepare an acidolysis reagent consisting of two protic acids (HBr and TFA), one Lewis acid (triethylbromide, TES-Br), and a carbocation scavenger (excess TES). Briefly, bromine (1 eq) was dissolved in TFA in a glass conical vial with gentle agitation, and the reaction mixture was cooled to 0 °C in an ice bath. Triethylsilane (1.9 eq) was then carefully added dropwise (note that the temperature increases and bubbling occurs) until the reddish-brown colour characteristic of bromine completely faded (30 min), as shown in the following reaction (Scheme 1):

The freshly prepared reagent was used to treat the cyclic benzyl-protected peptide. We first tested a solution of 9.8 wt% of generated HBr for 1 h (entry #2, Table 1), and 40% of the main product was obtained (Figure 2, #2). However, ESI-MS revealed that the major peak was a M-36 by-product, probably due to the dehydration of both threonine side chains, resulting in the formation of dehydrobutyrine (Dhb). To avoid the potential dehydration by-products originating from an excess of acid, the percentage of HBr and the reaction time were reduced. We found that a 2.8 wt% concentration of anhydrous HBr and a reaction time of 45 min (entry #4, Table 1) appeared to be the most effective acidolysis treatment, resulting in a peptide yield of 75% (Figure 2, #4). Applying the same treatment for 90 min (entry #5, Table 1), similar results were obtained (Figure 2, #5), whereas a 30-min treatment left polymyxin B_3_ partially protected (15% yield; entry #3, Table 1, Figure 2, #3).

**Figure 3:**
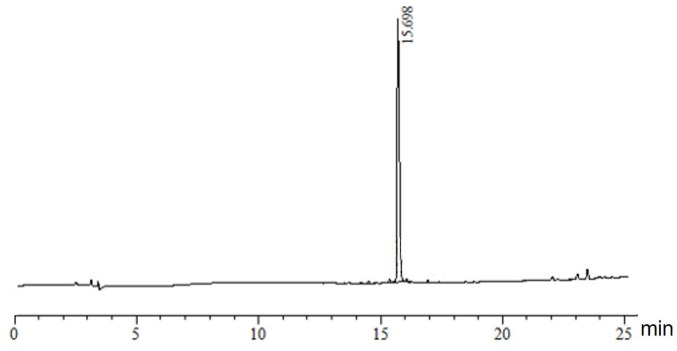
HPLC chromatogram of purified PxB_3_, using a linear gradient from 5% to 95% of B in 30 min; eluants, A: 0.045% TFA in H_2_O and B: 0.036% TFA in acetonitrile, detection at 220 nm.

Finally, the optimised deprotection conditions were applied on a larger scale. Starting with a batch of 254 mg of 2-CTC resin, a total amount of 339 mg of linearly protected peptide was obtained (ŋ_cleavage_ = 82%). After cyclisation, a crude of 257 mg of cyclic-protected peptide (ŋ_cyclization_ = 81%) was obtained and later treated with 100 mL of TFA/HBr/TES/Et_3_SiBr for 45 min. The TFA/HBr/TES/Et_3_SiBr solution was obtained by carefully reacting TFA/TES/Br_2_ (82.5:15:2.5, *v*/*v*/*v*) in an ice bath at 0 °C. The product was then precipitated in cold anhydrous diethyl ether, yielding a total amount of 184 mg (ŋ_acidolysis_= 92%) of a 72% pure solid analysed by HPLC, with a pale orange/off-white coloration that disappeared after purification by semi-preparative HPLC. The product was finally lyophilised, and 69 mg of the peptide in bromide form (47 mg of the free base compound) of >95% pure product were obtained (Figure 3). The characterisation was carried out by analytical HPLC and ESI-MS, and the global yield for the total synthesis of polymyxin B_3_ was 21%, a result that is comparable to other commonly used SPPS techniques [27,28].

To expand the scope of the HBr-containing cleavage reagent, we tested its capability to cleave peptides directly attached to a 4-methylbenzhydrylamine resin (MBHA). MBHA is a solid support that allows the preparation of C-terminal amidated peptides using a Boc/Bn protecting strategy. The release of carboxylic amides from the resin typically requires strong acids such as TMSOTf, TFMSA, or anhydrous HF. MBHA resin may be employed with the standard Fmoc/^t^Bu strategy if it is previously derivatised with a suitable bifunctional linker, such as the Rink-amide linker [29]. In this case, a standard TFA-based cleavage is generally required.

To further test the HBr novel reagent, two model C-terminal amidated antimicrobial peptides, namely, dusquetide and RR4 heptapeptide, were chosen (sequences shown in Table 2).

**Scheme 2:**
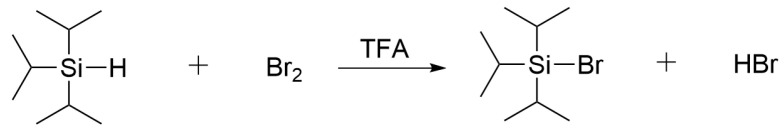
In situ hydrogen bromide generation by reaction of triisopropylsilane with bromine.

**Figure 4:**
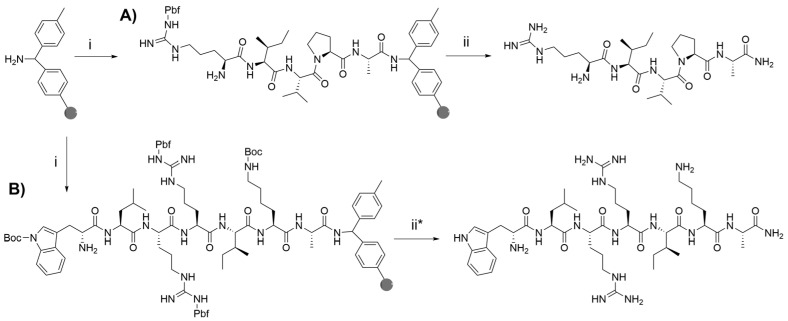
Complete scheme of the synthetic route of (**A**) dusquetide; (**B**) segments 8–14 of RR4. (i) Repetitive cycles of coupling and deprotection using a 3-fold molar excess of Fmoc-AA-OH/DIC/HOBt and 20% of piperidine in DMF (1 × 1 min, 2 × 10 min); (ii) TFA/HBr/Et_3_SiBr/TES, obtained by reaction at 0 °C of TFA:TES:Br_2_ (82.5:15:2.5) for 90 min at room temperature; (ii*) TFA/HBr/iP_3_SiBr/TIS, obtained by reaction at 0 °C of TFA:TIS:Br_2_ (82.5:15:2.5) for 90 min at room temperature.

Peptide amides were synthesised manually using the solid-phase technique, following standard Fmoc/^t^Bu procedures, directly on a benzhydryl (MBHA) resin (with no additional linker). The synthetic scheme is shown in Figure 4. On completion of the synthesis, dusquetide was detached from the resin by reaction with the in situ generated HBr reagent (HBr 2.8 wt%, entry #4, Table 1) under the optimised conditions previously described. However, according to other studies on peptides cleaved directly from MBHA resin using HBr or TMSBr [10,30], the reaction time was increased to 90 min to ensure the complete peptide release from the resin. Regarding the RR4 heptapeptide, the cleavage reagent was prepared with triisoproylsilane (TIS) instead of TES to prevent the indole ring reduction in the Trp residue (Scheme 2).

**Figure 5:**
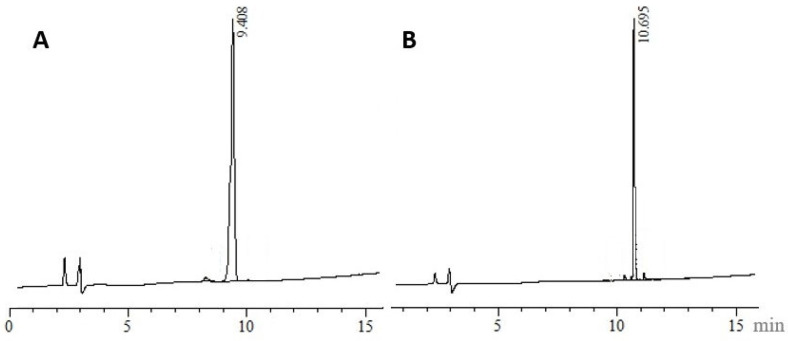
HPLC chromatograms of (**A**) dusquetide, (**B**) segment 8–14 of RR4 using a linear gradient from 5% to 95% of B over 30 min with an elution system of (**A**) 0.045% TFA in H_2_O and (**B**) 0.036% TFA in acetonitrile and processed at 220 nm.

It is well known that one of the most critical steps in peptide synthesis is the final cleavage, which involves the simultaneous deblocking of multiple protecting groups and detachment of the peptide from the resin. Deleterious side reactions may occur and modify susceptible residues, such as cysteine, methionine, or tryptophan. In this regard, we have taken the RR4 (8–14) peptide as an example of a challenging combination from the cleavage point of view since it contains one tryptophan and two arginine amino acids [31]. This combination of amino acids is known to be prone to several side reactions, including tryptophan oxidation, sulphonation, and alkylation. As shown in Figure 5, both dusquetide and RR4 heptapeptide were obtained with purities higher than 90% and cleavage yields of 87% and 82%, respectively. In addition, no evidence of indole reduction to the indoline or bromination was detected for RR4 heptapeptide, as shown by the absorption spectra of Trp residue presented in Figure S1 of the Supporting Information. Crudes were pale orange/off-white but the slight colour disappeared after a few cycles of lyophilisation in H_2_O/acetonitrile. Altogether, these results indicate that the novel acidolysis methodology is a highly promising approach to preparing peptide amides by Fmoc/^t^Bu chemistry directly on MBHA resin without the need for linkers.

Cationic peptides are generally obtained as trifluoroacetate salts after cleavage or HPLC purification if TFA is used as an eluent modifier. Currently, there are several options available for determining counterions in pharmaceutical salts. The most commonly used methods in the industry are ion chromatography (IC) and capillary electrophoresis (CE) [32]. However, there is still room for improvement in this analytical field. With the principal aim to characterise the full ionic composition of our synthetic antimicrobial peptides (subjected to the different acid treatments, i.e., HBr, TFA, and final aqueous HCl freeze drying), analytical techniques such as CHNS elemental analysis, titration, and mass spectrometry were explored.

### 3.2. CHNS Elemental Analysis

CHNS elemental analysis provides a means for the rapid determination of carbon, hydrogen, nitrogen, and sulphur in organic matrices. The Pregl-Dumas technique [33] consists of the combustion of the sample in an oxygen-rich environment in the presence of certain catalysts.

**Figure 6:**
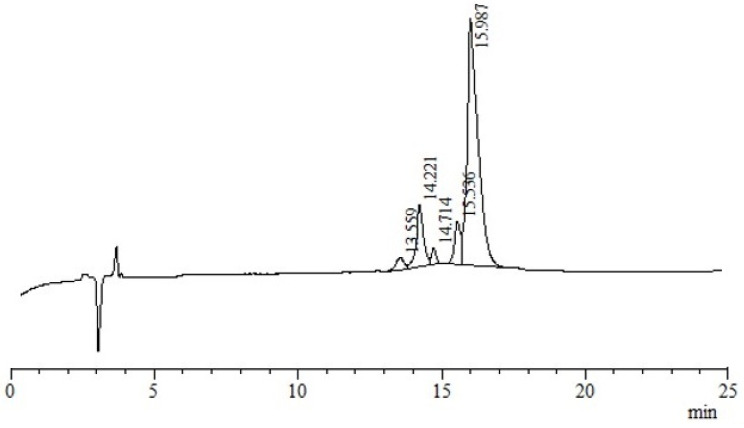
HPLC chromatogram of commercially available polymyxin B run at 1 mL/min using a linear gradient from 20% to 35% of B over 30 min with an elution system of A: 0.045% TFA in H_2_O and B: 0.036% TFA in acetonitrile and processed at 220 nm.

The technique is an easy, rapid, and cheap method to analyse the elemental composition of a pure peptide composed of C, H, N, and S. Therefore, it can only provide initial information on the presence of sulphate counterions in our case. The scope of this technique was explored using commercially available polymyxin B sulphate. As previously mentioned, polymyxin B is obtained by fermentation as a heterogeneous mixture of closely related lipopeptides (Figure 6) [14]. Therefore, the empirical formula of polymyxin B is not unique, and the analytical results can only yield an average value. According to the supplier, each polymyxin molecule is associated with two sulphate counterions and is assigned a formula of C_55_H_96_N_16_O_13_·2H_2_SO_4_ with a theoretical sulphur content of 4.63%. This corresponds to the major component polymyxin B1 and B1-Ile, which make up around 75–80% of the total content in polymyxin B [14]. The experimental result obtained was 4.22%, which reasonably adjusts to the molar content described by the supplier. However, this technique is not suitable to directly determine the counterion composition of our synthetic peptide batches as other anions such as chloride, bromide, or trifluoroacetate are involved.

### 3.3. Chloride Determination by Titration

Another interesting technique to analyse the counterion content of a sample is titration. Specifically, the direct titration method using silver nitrate is very useful for quantitatively analyse the chloride content of a sample.

To test the technique, a polymyxin B chloride sample was prepared from commercial polymyxin B sulphate as a proof of concept. Briefly, a polymyxin B sulphate sample was neutralised using equimolar amounts of KOH 0.1 M—4 equivalents corresponding to the two H_2_SO_4_ equivalents—to obtain the counterion free-base polymyxin B as a solid that precipitated in such neutral aqueous conditions. After washing the pellet several times with pure water and separating it by centrifugation, the obtained free-base polymyxin B was redissolved and lyophilised three times in 8 mM aqueous HCl. A crystalline polymyxin B solid was obtained as the hydrochloride salt.

The hydrochloride salt of polymyxin B (approximately 11 mgs) was analysed using the silver nitrate direct titration method, and a result of 12.70% (SD = 0.00087) chloride content was obtained (Table 3). This value correlates well with the theoretically calculated content of 12.92%, which corresponds to five chloride anions per polymyxin B natural mixture, corresponding to the five amino groups of the peptide. Hence, silver nitrate titration may be used to determine the chloride content of peptide samples, but it is hardly compatible with small-scale chemical syntheses due to the relatively large amounts needed for titration (1–10 mgs). Additionally, there is the potential risk that silver(I) ions may be complexed by peptides, leading to interference with the titration results, particularly, when the peptides contain histidine, tryptophan, methionine, or cysteine [34,35,36]. Hence, the synthetically obtained peptides were not analysed by this method.

### 3.4. Electrospray Ionisation Mass Spectrometry (nanoESI-MS and ESI-MS)

NanoESI and ESI-MS experiments are typically adjusted and designed to minimise all non-volatile counterions and adducts before MS because cluster formation usually reduces analyte signals and complicates mass spectra. However, ion complexes can be transferred intact to the gas phase and detected either in positive or negative polarities by modulating parameters such as the capillary temperature, extraction voltages, and/or source pressure. In this case, clusters of the protonated peptide with its associated counter ions were intended to be observed [37].

To explore the scope of this technique, three samples collected at different stages of polymyxin B_3_ synthesis were analysed: (1) a crude sample collected after treatment with the HBr acidolysis reagent; (2) polymyxin B_3_ obtained by HPLC purification using 0.1% TFA eluents; and (3) HPLC-purified polymyxin B_3_ obtained by lyophilisation with an excess of aqueous HCl as described above.

**Figure 7:**
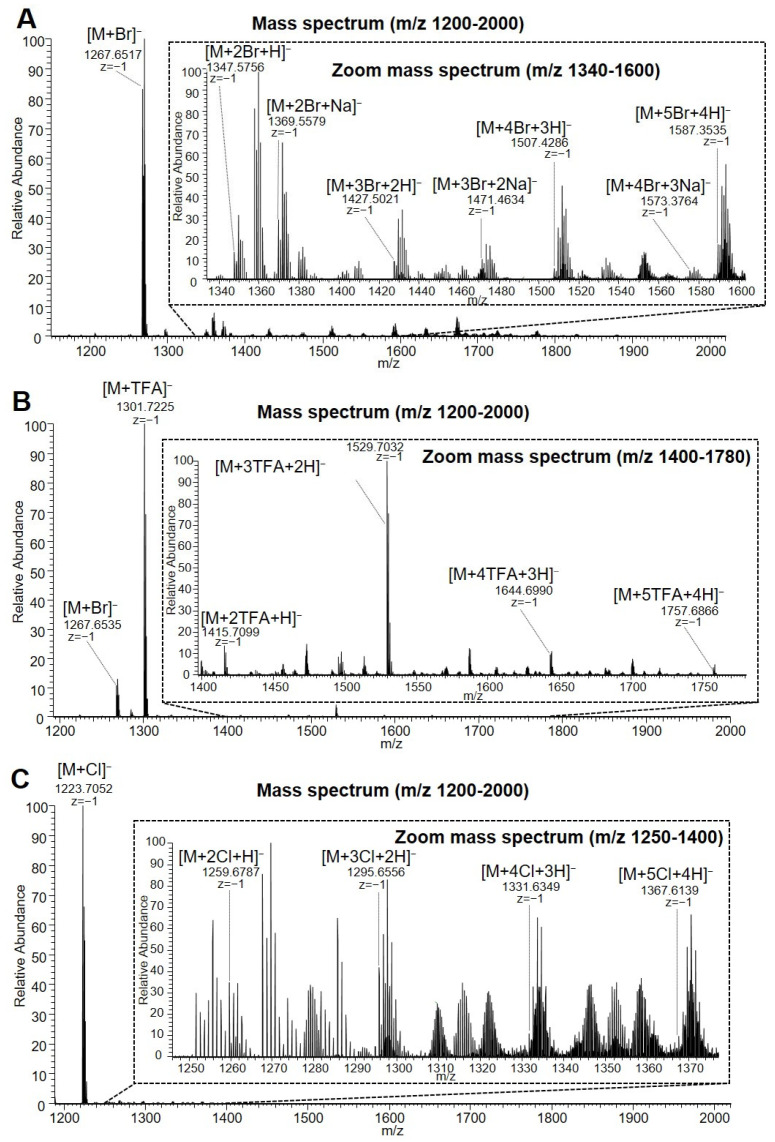
Spectra obtained by nanoESI-HR MS in the negative mode for samples: (**A**) PxB_3_ after the in situ-generated HBr acidolysis method; (**B**) purified PxB_3_; and (**C**) PxB_3_, obtained after the aqueous HCl exchange by lyophilization. “z” indicates the charge of the non-covalent ion complexes detected.

Samples were first analysed at high resolution (120 k) and negative polarity in an Orbitrap Fusion Lumos™ Tribrid (Thermo Scientific) mass spectrometer with a chip-based nanoelectrospray source (Advion Triversa Nanomate from Advion BioSciences, Ithaca, NY, USA). The obtained nanoESI spectra are shown in Figure 7, and the detailed isotopic pattern of each species compared with its theoretical spectrum is shown in Supplementary Information.

In the first analysed sample (Figure 7A), corresponding to the reaction crude after acidic treatment with HBr, the species [M + Br]^−^, [M + 2Br + H]^−^, [M + 3Br + 2H]^−^, [M + 4Br + 3H]^−^ and [M + 5Br + 4H]^−^ were detected, with [M + Br]^−^ the main species. Clusters of PxB_3_ with TFA were not observed, even though TFA is the major component in the acidolysis reagent.

The second studied polymyxin B_3_ sample had been purified using conventional gradients of acetonitrile-water-0.1% trifluoroacetic acid. After purification in these conditions, the bromide counterions appeared to be exchanged by trifluoroacetates, and the purified peptide was obtained as the trifluoroacetate salt (Figure 7B). The species [M + TFA]^−^, [M + 2TFA + H]^−^, [M + 3TFA + 2H]^−^, [M + 4TFA + 3H]^−^ and [M + 5TFA + 4H]^−^ were observed, whereas a unique peptide cluster with bromide was also detected [M + Br]^−^, with [M + TFA]^−^, [M + Br]^−^ and [M + 3TFA + H]^−^ the most abundant species. Apparently, there were no mixed ionic species such as [M + Br + TFA + H]^−^.

Finally, the hydrochloride salt of polymyxin B_3_ was analysed (Figure 7C). The sample was obtained after three cycles of peptide dissolution and lyophilisation in the presence of an excess of HCl (8 mM). In this case, the detected species were [M + Cl]^−^, [M + 2Cl + H]^−^, [M + 3Cl + 2H]^−^, [M + 4Cl + 3H]^−^ and [M + 5Cl + 4H]^−^, with [M + Cl]^−^ the most abundant specie. No traces of trifluoroacetate salts were detected, indicating that the counterion exchange was completed.

After reviewing the results, we assessed the feasibility of implementing product quality controls by MS in a more accessible mass to achieve an effective balance between product quality and cost. We aimed to explore a quadrupole-resolution instrument tuned to detect negative ion clusters. For that purpose, a Waters Micromass ZQ 4000 spectrometer was employed.

**Figure 8:**
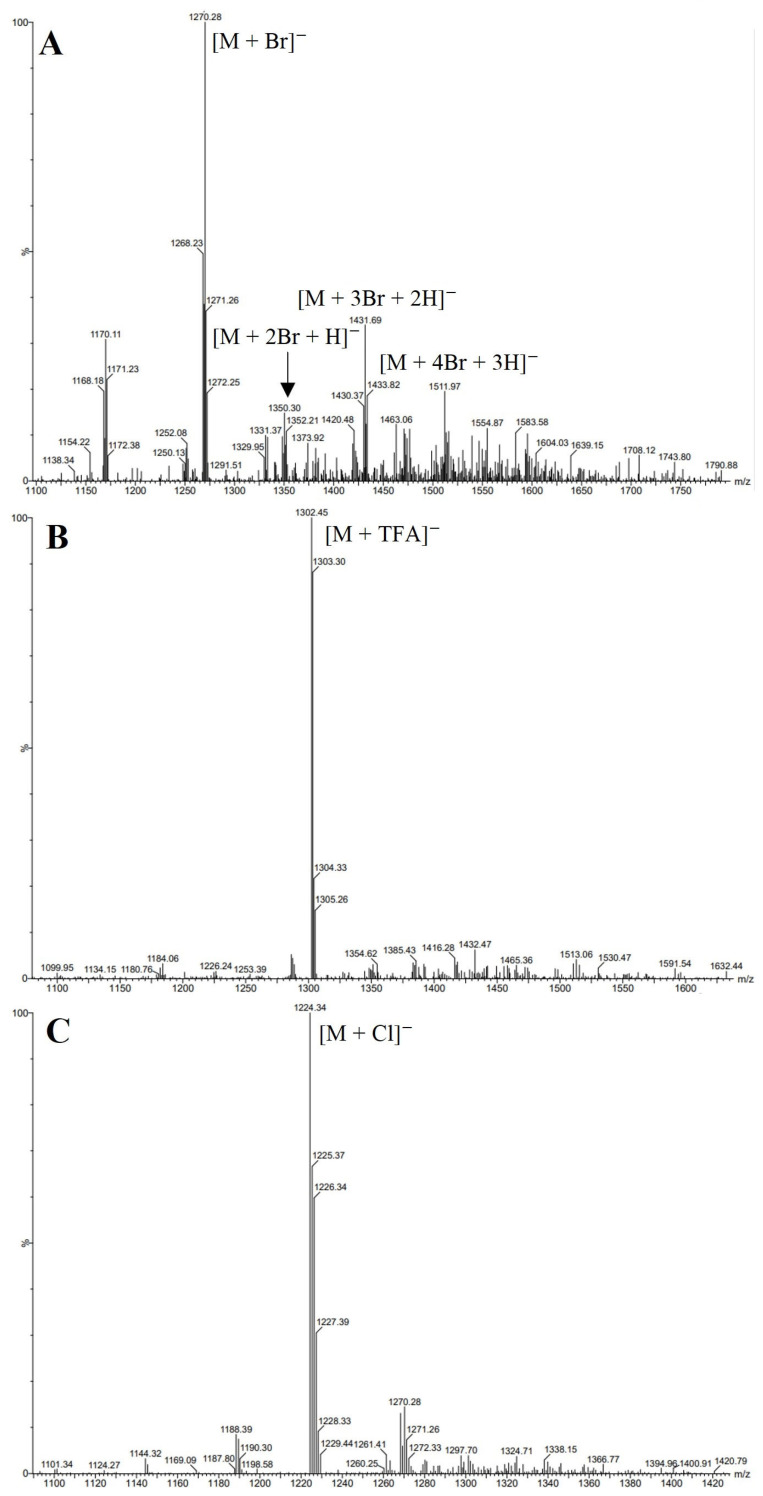
Spectra obtained by ESI-MS in the negative mode for samples: (**A**) reaction crude of synthetic PxB_3_ after HBr deprotection treatment; (**B**) purified PxB_3_; and (**C**) PxB_3_ hydrochloride salt.

Remarkably, bromide clusters were clearly identified, making it possible to assign species such as [M + Br]^−^, [M + 2Br + H]^−^, [M + 3Br + 2H]^−^ and [M + 4Br + 3H]^−^. As before, [M + TFA]^−^ and [M + Cl]^−^ clusters were also identified in the corresponding samples (Figure 8).

**Figure 9:**
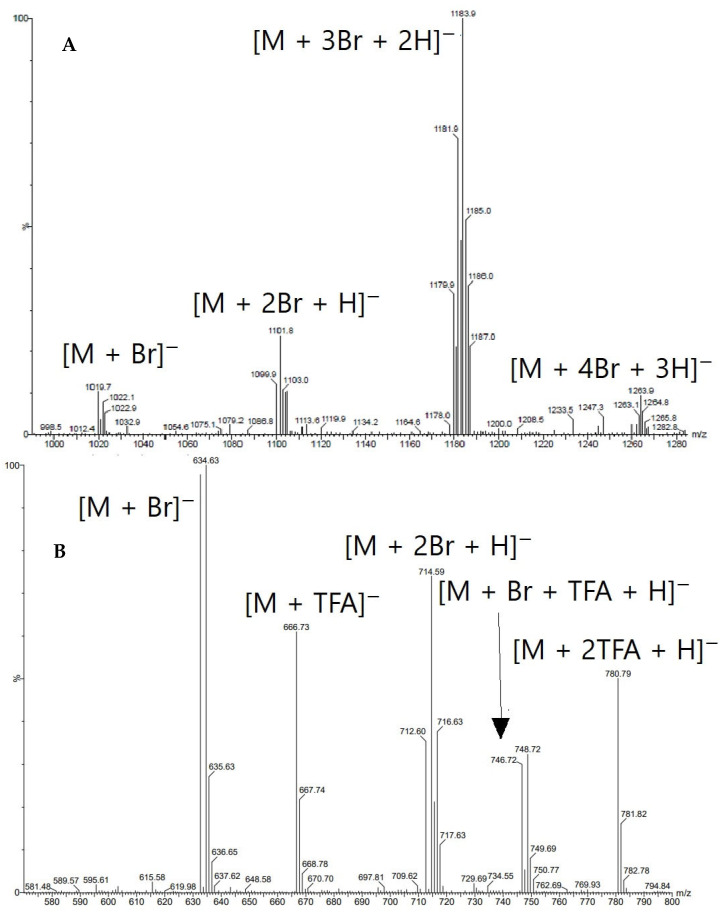
Spectra obtained by ESI-MS in the negative mode for samples: (**A**) crude RR4; (**B**) crude dusquetide. The MS spectra correspond to samples whose HPLC profile can be found in Figure 5.

The reaction crudes of dusquetide and RR4 heptapeptide were analysed through ESI-MS to determine their ion composition. For RR4, the following species were detected: [M + Br]^−^, [M + 2Br + H]^−^, [M + 3Br + 2H]^−^, and [M + 4Br + 3H]^−^, which was consistent with the four basic groups present in the sequence. The main species detected was [M + 3Br + 2H]^−^. Regarding dusquetide, the clusters [M + Br]^−^, [M + TFA]^−^, [M + 2Br + H]^−^, [M + 2TFA + H]^−^, and [M + Br + TFA + H]^−^ were detected as expected. In this last case, mixed clusters of bromide and trifluoroacetate were detected. The mass spectra for both peptide crudes are shown in Figure 9. ESI-MS spectra confirming the *m*/*z* in positive mode for all synthetic peptides are shown in the Supporting Information.

In conclusion, we have found that negative polarity nanoESI-MS and ESI-MS permit fully characterising the counterion composition of cationic peptides in properly tuned mass spectrometers. More precisely, it allowed determining the whole salt composition of purified synthetic samples of polymyxin B_3_ as well as purified synthetic RR4 and dusquetide peptides after treatment using different acidolysis and purification conditions. Both MS experiments, at high and low resolution, performed on instruments with different sensitivity capabilities, enabled the detection of the main species in each sample. Furthermore, the amount of sample required for routine analysis is small (around 0.1 mg) and is compatible with the standard peptide synthesis at the laboratory scale. This is unlike the other two techniques discussed above: CHNS elemental analysis and chloride titration, which require much higher amounts of sample.

### 3.5. Antimicrobial Activity

As proof of biological activity, the minimum inhibitory concentration (MIC) of the different polymyxin salts was evaluated [38]. The assay consisted of serial two-fold dilutions from 0.06 to 32 μg·mL^−1^ of peptide, which were incubated with a standardised number of microorganisms (10^6^ CFU·mL^−1^) at 37 °C for 18 h. The MIC was determined visually based on turbidity as the lowest peptide concentration inhibiting bacterial growth. The commercially available PxB sulphate and the synthetic PxB_3_ forms, including trifluoroacetate, hydrochloride, and hydrobromide salts, were evaluated for their antibacterial activity against the Gram-negative *Pseudomonas aeruginosa* by determining their minimum inhibitory concentration (MIC). All compounds showed the same antimicrobial activities, with MIC values of 1 μg·mL^−1^ (Table 4). Therefore, it can be concluded that the presence of different counterions has no significant impact on the antimicrobial activity of polymyxins.

## 4. Conclusions

A novel anhydrous hydrogen bromide cleavage reagent for the deprotection of benzyl-type protecting groups and detachment of peptides from 4-methylbenzhydryl amine resins has been described and optimised. This methodology involves an in situ-generated reagent composed of HBr and a silyl bromide prepared by a reaction of bromine and a silane in trifluoroacetic acid (i.e., TFA/TES/Br_2_ (82.5:15:2.5, *v*/*v*/*v*; at 0 °C, careful handling). The novel reagent allowed the full benzyl ether and carbamate deprotection as demonstrated in the synthesis of the cyclic lipopeptide polymyxin B_3_ using a Fmoc/Bn/tBu scheme of protection on a chlorotrityl chloride resin. Altogether, the total synthesis of polymyxin B_3_ gave a satisfactory global yield of 21% after HPLC purification (>95% purity), comparable to common SPPS techniques. The novel method was also effective for the cleavage and deprotection of two model peptides (dusquetide and RR4 heptapeptide) assembled following a Fmoc/tBu protection scheme directly attached to 4-methylbenzhydrylamine (MBHA) resins with no need of using any intermediate linker such as the Rink one. The two model peptides were obtained with excellent cleavage yields (87 and 82%, respectively) and crude purities around 80 and 95%, respectively. The RR4 heptapeptide is a particularly challenging peptide from the acidolysis point of view as it contains two arginines and a tryptophan within the sequence. Altogether, we believe this novel methodology is a highly promising approach to the solid-phase synthesis of peptides and expect that its full development will be accomplished in future works.

Additionaly, different techniques for the full characterisation of the peptide salt composition (bromide, trifluoroacetate, sulphate, and chloride) have been described. CHNS elemental analysis and titration gave quantitative information about the ionic content of polymyxin B samples, which contain counterions such as sulphate or chloride. However, they presented some drawbacks, such as the relatively large amount of sample required, which is hardly compatible with small-scale laboratory synthesis, and the limited number of counterions that could be detected. Finally, electrospray ionisation mass spectrometry (ESI-MS and nanoESI) was found to be an easy, rapid, and sensitive tool for fully characterising the counterions present in cationic peptides. It is possible to determine the whole ionic composition of the peptides at different stages (crude, HPLC-purified, or exchanged) using this method. All forms of polymyxin retained their antibacterial activity against *Pseudomonas aeruginosa*, a pathogenic bacteria considered a serious health threat by the European and US Centers for Disease Control and Prevention (ECDC and CDC).

## Figures and Tables

**Table 1:** Optimisation of the acidolysis reagent using the cyclic benzyl-protected peptide PxB_3_ synthesised following a Fmoc/Bn/^t^Bu protection strategy as described in Figure 1. Crude purity was established by the integration of the corresponding HPLC peak.

	Acidic Mixture	HBr wt%	Volume Ratio	Time	Crude Purity
#1	TFA/TMSCl/TES/HBr 33% of AcOH	1.8	64.2:25.8:5:5	2 h	6%
#2	TFA/TES/Br_2_ (TFA/HBr/Et_3_SiBr/TES)	9.8	47.5:45:7.5	1 h	0%
#3	TFA/TES/Br_2_ (TFA/HBr/Et_3_SiBr/TES)	2.8	82.5:15:2.5	30 min	60%
#4	TFA/TES/Br_2_ (TFA/HBr/Et_3_SiBr/TES)	2.8	82.5:15:2.5	45 min	75%
#5	TFA/TES/Br_2_ (TFA/HBr/Et_3_SiBr/TES)	2.8	82.5:15:2.5	90 min	71%

**Table 2:** Sequences of dusquetide and RR4 antimicrobial peptides.

Peptide	Sequence
Dusquetide	*H*-Arg-Ile-Val-Pro-Ala-*NH*_2_
RR4 (8–14)	*H*-Trp-Leu-Arg-Arg-Ile-Lys-Ala-*NH*_2_

**Table 3:** Summary of the chloride determination by titration of prepared polymyxin B·5HCl.

Amount of Sample [mg]	Consumption of AgNO_3_ 0.01 M [mL]	Cl Content [%]	Mean	Standard Deviation
3.87	1.4012	12.69	12.70	0.00087
3.22	1.1674	12.67		
3.59	1.3069	12.74		

**Table 4:** Antibacterial activity of commercially available and synthetic salts of polymyxins.

Polymyxin Salts	MIC [μg·mL^−1^] *Pseudomonas* *aeruginosa* ATCC 27853
Polymyxin B, sulfate salt, commercial	1
Polymyxin B_3_, trifluoroacetate salt, synthetic	1
Polymyxin B_3_, hydrochloride salt, synthetic	1
Polymyxin B_3_, hydrobromide salt, synthetic	1

## Data Availability

Not applicable.

## Supplementary Materials

The following supporting information can be downloaded at: https://www.mdpi.com/article/10.3390/pharmaceutics15041310/s1. Table S1. Detected polymyxin ion clusters obtained by nanoESI-HR MS in negative mode. Table S2: Detected polymyxin, RR4, or dusquetide ion clusters obtained by a quadrupole-resolution instrument tuned to detect negative ion clusters. Table S3. The ESI-MS characterisation (positive mode) was used for the synthetic polymyxins B3, RR4, and dusquetide peptides obtained in a Waters ESI-MS Micromass ZQ 4000 spectrometer. Figure S1. Abortion spectrum of tryptophan. A) H-Trp(OAll)·HCl; B) RR4 heptapeptide after cleavage with TFA/TES/Br_2_ (82.5:15:2.5, *v*/*v*/*v*) for 45 min. Figure S2: Experimental and theoretical isotopic distribution of the peak corresponding to [M + Br]^−^, C_55_H_96_O_13_N_16_Br. Figure S3: Experimental and theoretical isotopic distribution of the peak corresponding to [M + 2Br + H]^−^, C_55_H_97_O_13_N_16_Br_2_. Figure S4. Experimental and theoretical isotopic distribution of the peak corresponding to [M + 3Br + 2H]^−^, C_55_H_98_O_13_N_16_Br_3_. Figure S5. Experimental and theoretical isotopic distribution of the peak corresponding to [M + 4Br + 3H]^−^, C_55_H_99_O_13_N_16_Br_4_. Figure S6. Experimental and theoretical isotopic distribution of the peak corresponding to [M + 5Br + 4H]^−^, C_55_H_100_O_13_N_16_Br_5_. Figure S7. Experimental and theoretical isotopic distribution of the peak corresponding to [M + Br]^−^, C_55_H_96_O_13_N_16_Br. Figure S8. Experimental and theoretical isotopic distribution of the peak corresponding to [M + TFA]^−^, C_57_H_96_O_15_N_16_F_3_. Figure S9. Experimental and theoretical isotopic distribution of the peak corresponding to [M + 2TFA + H]^−^, C_59_H_97_O_17_N_16_F_6_. Figure S10. Experimental and theoretical isotopic distribution of the peak corresponding to [M + 3TFA + 2H]^−^, C_61_H_98_O_19_N_16_F_9_. Figure S11. Experimental and theoretical isotopic distribution of the peak corresponding to [M + 4TFA + 3H]^−^, C_63_H_99_O_21_N_16_F_12_. Figure S12. Experimental and theoretical isotopic distribution of the peak corresponding to [M + 5TFA + 4H]^−^, C_65_H_100_O_23_N_16_F_15_. Figure S13. Experimental and theoretical isotopic distribution of the peak corresponding to [M + Cl]^−^, C_55_H_96_O_13_N_16_Cl. Figure S14. Experimental and theoretical isotopic distribution of the peak corresponding to [M + 2Cl + H]^−^, C_55_H_97_O_13_N_16_Cl_2_. Figure S15. Experimental and theoretical isotopic distribution of the peak corresponding to [M + 3Cl + 2H]^−^, C_55_H_98_O_13_N_16_Cl_3_. Figure S16. Experimental and theoretical isotopic distribution of the peak corresponding to [M + 4Cl + 3H]^−^, C_55_H_99_O_13_N_16_Cl_4_. Figure S17. Experimental and theoretical isotopic distribution of the peak corresponding to [M + 5Cl + 4H]^−^, C_55_H_100_O_13_N_16_Cl_5_. Figure S18. Spectra obtained by ESI-MS in positive mode for polymyxin B_3_ peptide. Figure S19. Spectra obtained by ESI-MS in positive mode for RR4 heptapeptide. Figure S20. Spectra obtained by ESI-MS in positive mode for the dusquetide peptide.
