# Supplementary material for: Linker-Free Synthesis of Antimicrobial Peptides Using a Novel Cleavage Reagent: Characterisation of the Molecular and Ionic Composition by nanoESI-HR MS
Source: Pharmaceutics. 2023 Apr 21;15(4):1310. doi: 10.3390/pharmaceutics15041310 (PMC10141159; doi:10.3390/pharmaceutics15041310)
Supplement: Supplementary file 1 [file pharmaceutics-15-01310-s001.zip › pharmaceutics-2311152-supplementary.pdf]

Supplementary Information

Table S1. Detected polymyxin ion clusters obtained by nanoESI-HR MS in negative mode.

Compound	Chemical formula	Theoretical mass	m/z	Delta (ppm)
**[PxB$_3$+Br]$^-$**	$C_{55}H_{96}O_{13}N_{16}Br$	1267.65316	1267.65166	-1.19
**[PxB$_3$+2Br+H]$^-$**	$C_{55}H_{97}O_{13}N_{16}Br_2$	1347.57933	1347.57566	-2.72
**[PxB$_3$+3Br+2H]$^-$**	$C_{55}H_{98}O_{13}N_{16}Br_3$	1427.50549	1427.50214	-2.35
**[PxB$_3$+4Br+3H]$^-$**	$C_{55}H_{99}O_{13}N_{16}Br_4$	1507.43165	1507.42861	-2.02
**[PxB$_3$+5Br+4H]$^-$**	$C_{55}H_{100}O_{13}N_{16}Br_5$	1587.35781	1587.35352	-2.70
**[PxB$_3$+2Br+Na]$^-$**	$C_{55}H_{96}O_{13}N_{16}Br_2Na$	1369.56078	1369.55790	-2.10
**[PxB$_3$+3Br+2Na]$^-$**	$C_{55}H_{96}O_{13}N_{16}Br_3Na_2$	1471.46889	1471.46340	-3.73
**[PxB$_3$+4Br+3Na]$^-$**	$C_{55}H_{96}O_{13}N_{16}Br_4Na_3$	1573.37699	1573.37640	-0.37
**[PxB$_3$+Br]$^-$**	$C_{55}H_{96}O_{13}N_{16}Br$	1267.65316	1267.65352	0.28
**[PxB$_3$+TFA]$^-$**	$C_{57}H_{96}O_{15}N_{16}F_3$	1301.71986	1301.72247	2.00
**[PxB$_3$+2TFA+H]$^-$**	$C_{59}H_{97}O_{17}N_{16}F_6$	1415.71273	1415.70990	-2.00
**[PxB$_3$+3TFA+2H]$^-$**	$C_{61}H_{98}O_{19}N_{16}F_9$	1529.70559	1529.70270	-1.89
**[PxB$_3$+4TFA+3H]$^-$**	$C_{63}H_{99}O_{21}N_{16}F_{12}$	1643.69846	1643.69595	-1.52
**[PxB$_3$+5TFA+4H]$^-$**	$C_{65}H_{100}O_{23}N_{16}F_{15}$	1757.69132	1757.68656	-2.71
**[PxB$_3$+Cl]$^-$**	$C_{55}H_{96}O_{13}N_{16}Cl$	1223.70368	1223.70517	1.22
**[PxB$_3$+2Cl+H]$^-$**	$C_{55}H_{97}O_{13}N_{16}Cl_2$	1259.68036	1259.67870	-1.31
**[PxB$_3$+3Cl+2H]$^-$**	$C_{55}H_{98}O_{13}N_{16}Cl_3$	1295.65703	1295.65562	-1.09
**[PxB$_3$+4Cl+3H]$^-$**	$C_{55}H_{99}O_{13}N_{16}Cl_4$	1331.63371	1331.63487	0.87
**[PxB$_3$+5Cl+4H]$^-$**	$C_{55}H_{100}O_{13}N_{16}Cl_5$	1367.61039	1367.61386	2.54

Table S2: Detected polymyxin, RR4, or dusquetide ion clusters obtained by a quadrupole-resolution instrument tuned to detect negative ion clusters.

Compound	Chemical formula	Theoretical mass	m/z	Delta (ppm)
**[PxB$_3$+Br]$^-$**	$C_{55}H_{96}O_{13}N_{16}Br$	1267.65316	1268.23	455.05
**[PxB$_3$+2Br+H]$^-$**	$C_{55}H_{97}O_{13}N_{16}Br_2$	1347.57933	1348.37	586.73
**[PxB$_3$+3Br+2H]$^-$**	$C_{55}H_{98}O_{13}N_{16}Br_3$	1427.50549	1430.37	2006.65
**[PxB$_3$+4Br+3H]$^-$**	$C_{55}H_{99}O_{13}N_{16}Br_4$	1507.43165	1511.97	3010.65
**[PxB$_3$+TFA]$^-$**	$C_{57}H_{96}O_{15}N_{16}F_3$	1301.71986	1302.45	560.90
**[PxB$_3$+Cl]$^-$**	$C_{55}H_{96}O_{13}N_{16}Cl$	1223.70368	1224.34	519.00
**[RR4+Br]$^-$**	$C_{44}H_{76}N_{16}O_7Br$	1019.52663	1019.7	170.05
**[RR4+2Br+H]$^-$**	$C_{44}H_{77}N_{16}O_7Br_2$	1099.45279	1099.9	406.76
**[RR4+3Br+2H]$^-$**	$C_{44}H_{78}N_{16}O_7Br_3$	1179.37895	1179.9	441.80
**[RR4+4Br+3H]$^-$**	$C_{44}H_{79}N_{16}O_7Br_4$	1259.30511	1263.1	3013.48
**[Dusquetide+Br]$^-$**	$C_{25}H_{47}N_9O_5Br$	632.28835	632.63	540.34
**[Dusquetide+2Br+H]$^-$**	$C_{25}H_{48}N_9O_5Br_2$	712.21451	712.60	541.26
**[Dusquetide+TFA]$^-$**	$C_{27}H_{47}N_9O_7F_3$	666.35506	666.73	562.67
**[Dusquetide+2TFA+2H]$^-$**	$C_{29}H_{48}N_9O_9F_6$	780.34792	780.79	566.52
**[Dusquetide+Br+TFA+2H]$^-$**	$C_{27}H_{48}N_9O_7F_3Br$	746.28122	746.72	587.96

Table S3: The ESI-MS characterisation (positive mode) was used for the synthetic polymyxins B3, RR4, and dusquetide peptides obtained in a Waters ESI-MS Micromass ZQ 4000 spectrometer.

Compound	Chemical formula	Theoretical mass	m/z	Delta (ppm)
**[PxB$_3$+H]$^+$**	$C_{55}H_{97}O_{13}N_{16}$	1189.74210	1190.11	309.23
**[PxB$_3$+2H]$^{2+}$**	$C_{55}H_{98}O_{13}N_{16}$	595.37497	595.68	512.33
**[RR4+H]$^+$**	$C_{44}H_{77}O_7N_{16}$	941.61611	941.8	6567.32
**[RR4+2H]$^{2+}$**	$C_{44}H_{78}O_7N_{16}$	471.31197	471.1	-449.74
**[RR4+3H]$^{3+}$**	$C_{44}H_{79}O_7N_{16}$	314.54392	314.6	178.29
**[Dusquetide+H]$^+$**	$C_{25}H_{48}N_9O_5$	554.37784	554.64	472.89

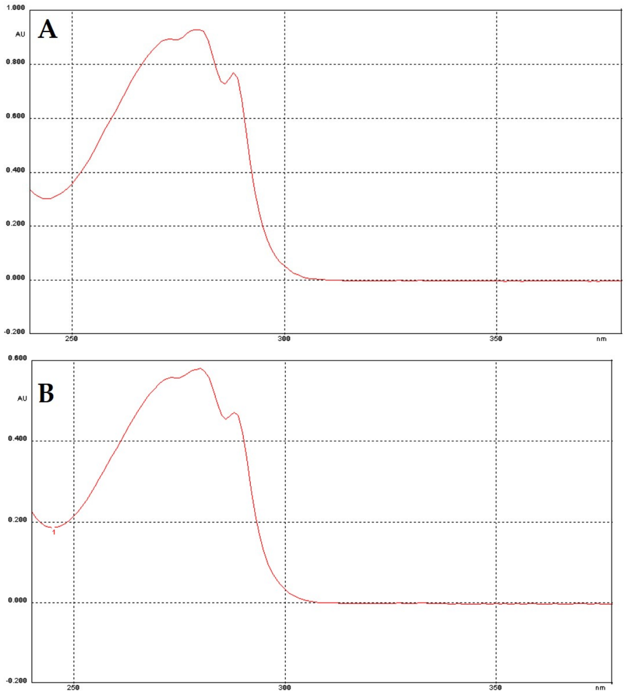

Figure S1: Abortion spectrum of tryptophan. A) H-Trp(OAll)·HCl; B) RR4 heptapeptide after cleavage with TFA/TES/Br_2 (82.5:15:2.5, v/v/v) for 45 minutes.

The following images correspond to the study of the isotopic distribution of the different species obtained by ESI-HR MS of synthetic PxB_3:

PxB_3 after cleavage with HBr (a detailed study of Figure 7A)

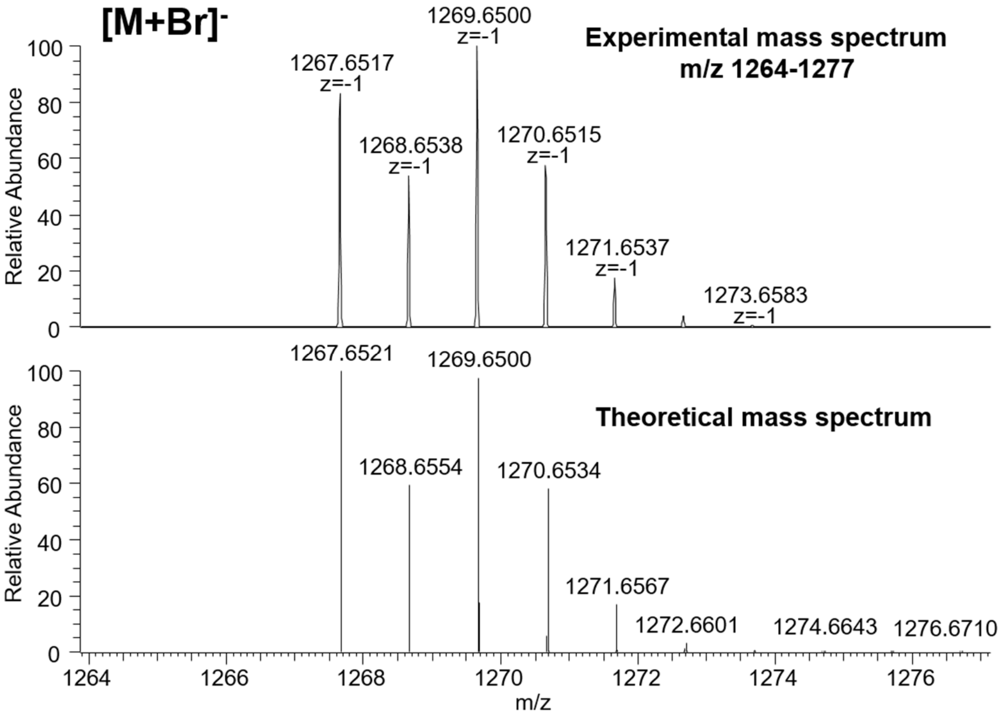

Figure S2: Experimental and theoretical isotopic distribution of the peak corresponding to $[M + Br]^-$, $C_{55}H_{96}O_{13}N_{16}Br$.

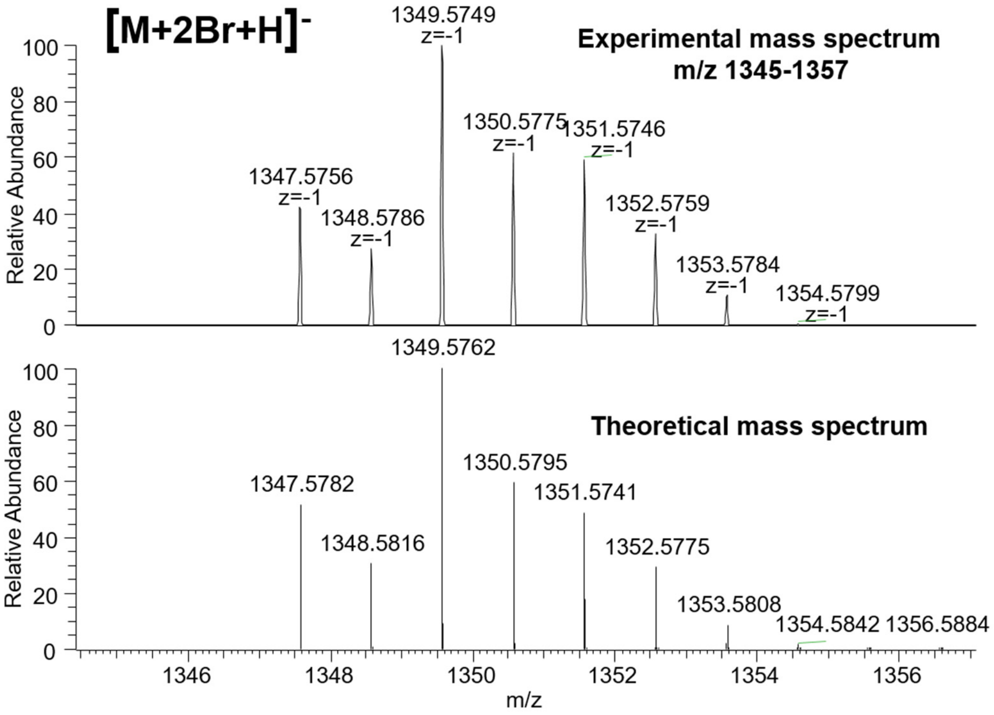

Figure S3: Experimental and theoretical isotopic distribution of the peak corresponding to $[M + 2Br + H]^-$, $C_{55}H_{97}O_{13}N_{16}Br_2$.

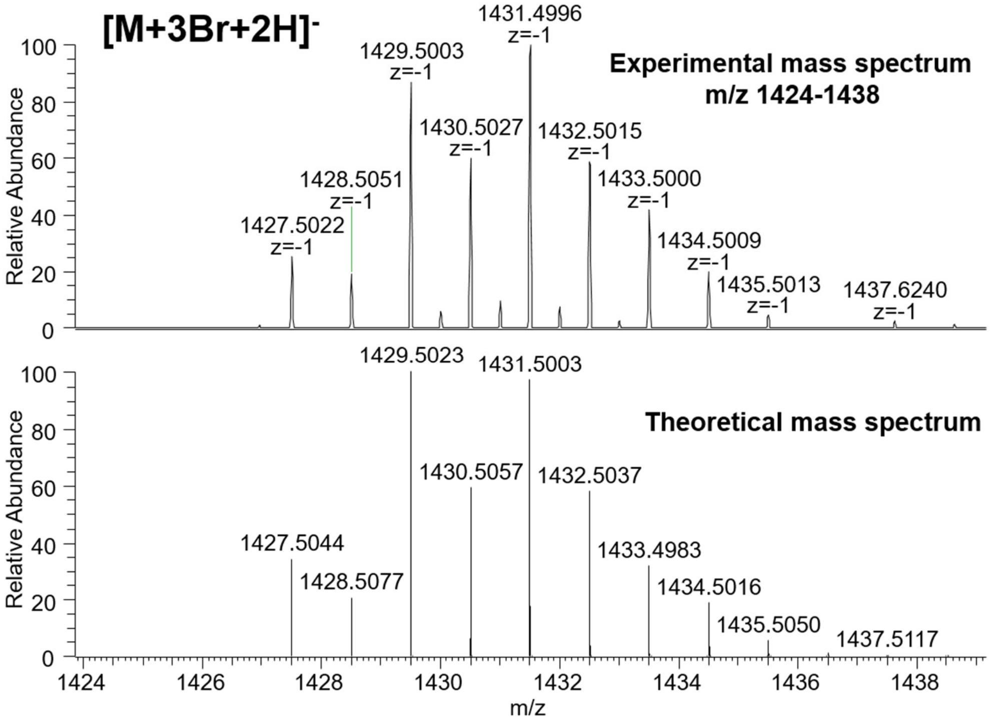

Figure S4: Experimental and theoretical isotopic distribution of the peak corresponding to $[M + 3Br + 2H]^-$, $C_{55}H_{98}O_{13}N_{16}Br_3$.

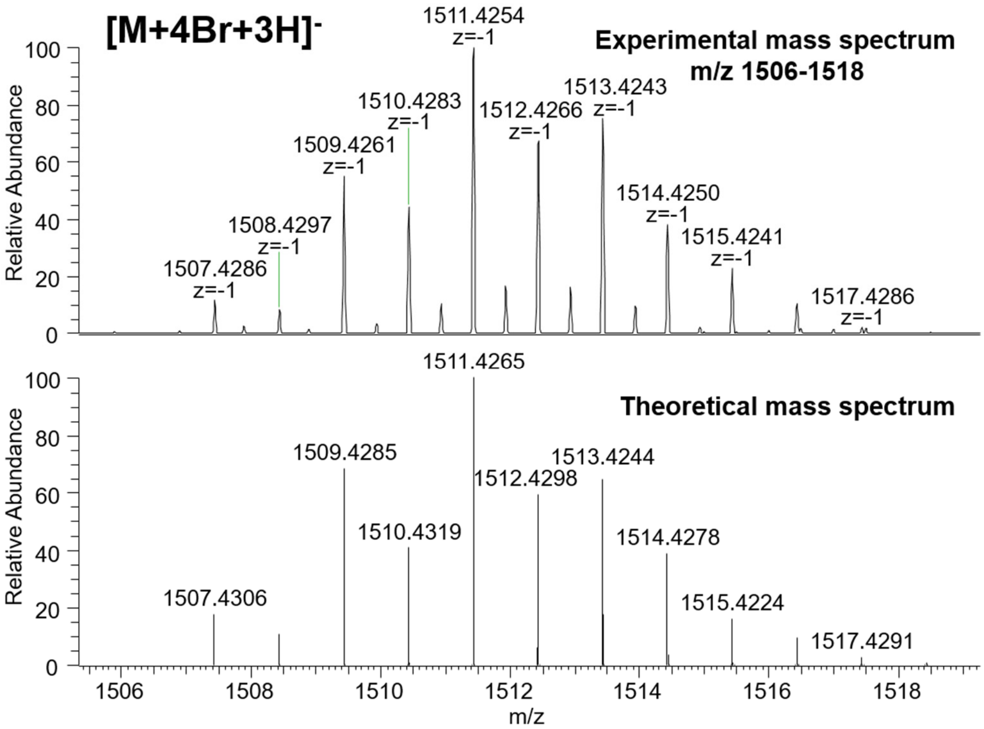

Figure S5: Experimental and theoretical isotopic distribution of the peak corresponding to $[M + 4Br + 3H]^-$, $C_{55}H_{99}O_{13}N_{16}Br_4$.

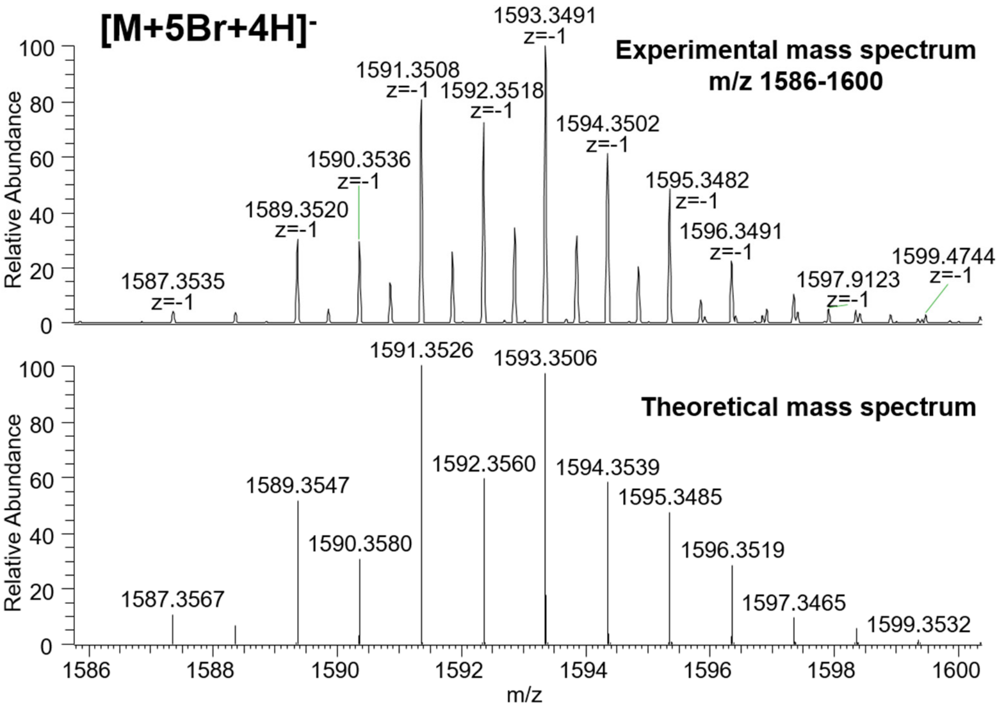

Figure S6: Experimental and theoretical isotopic distribution of the peak corresponding to $[M + 5Br + 4H]^-$, $C_{55}H_{100}O_{13}N_{16}Br_5$.

PxB$_3$ after purification in presence of 0.1 % TFA (a detailed study of Figure 7B):

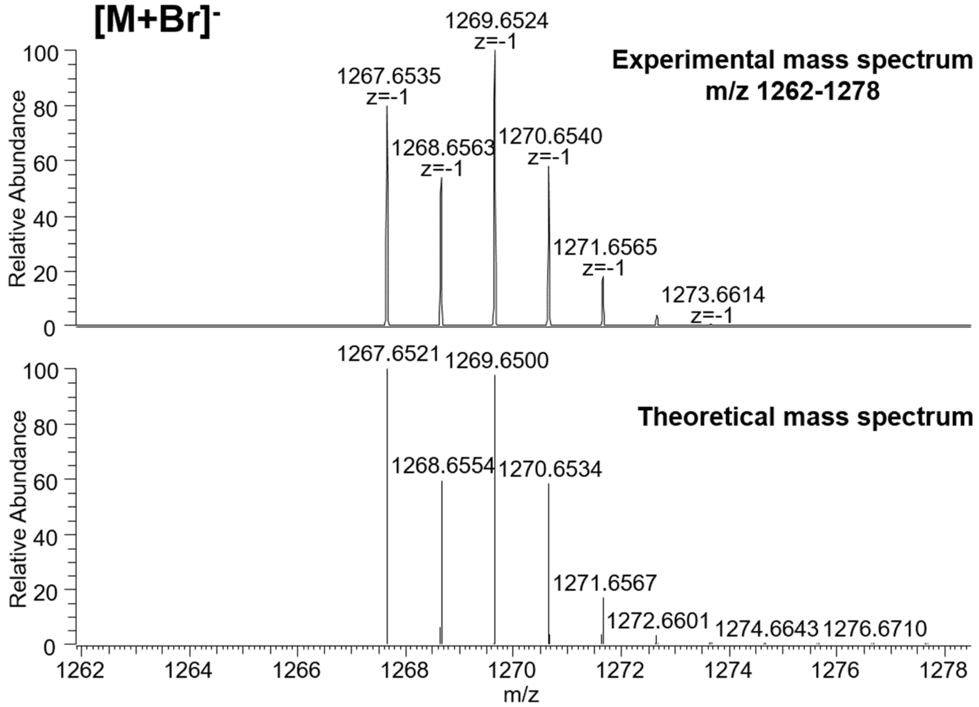

Figure S7: Experimental and theoretical isotopic distribution of the peak corresponding to $[M + Br]^-$, $C_{55}H_{96}O_{13}N_{16}Br$.

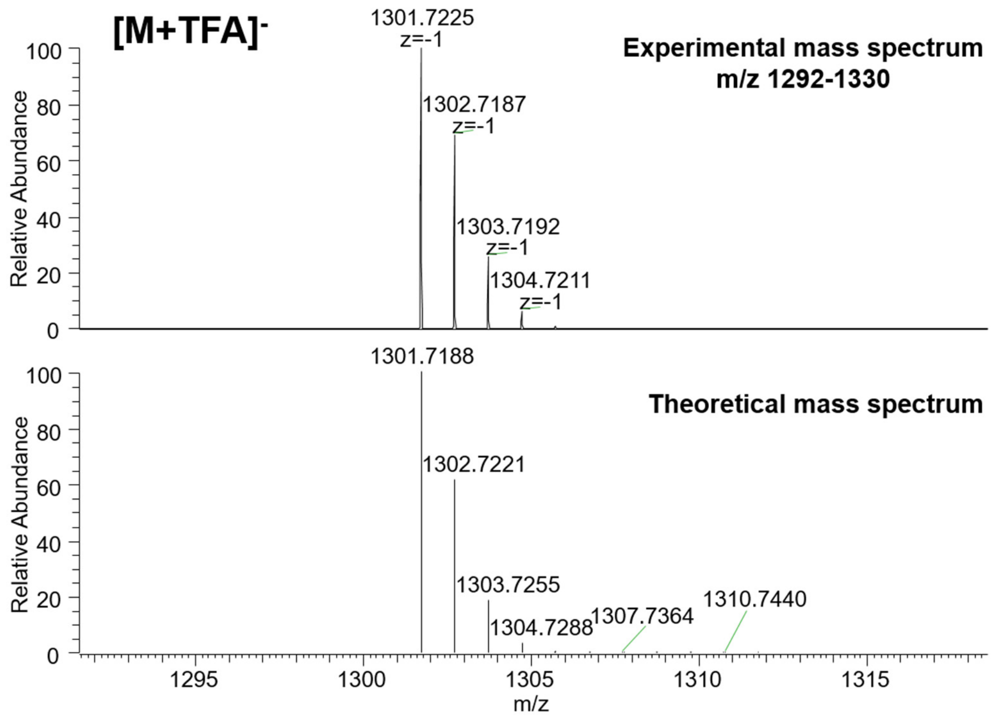

Figure S8: Experimental and theoretical isotopic distribution of the peak corresponding to [M + TFA]-, $C_{57}H_{96}O_{15}N_{16}F_3$.

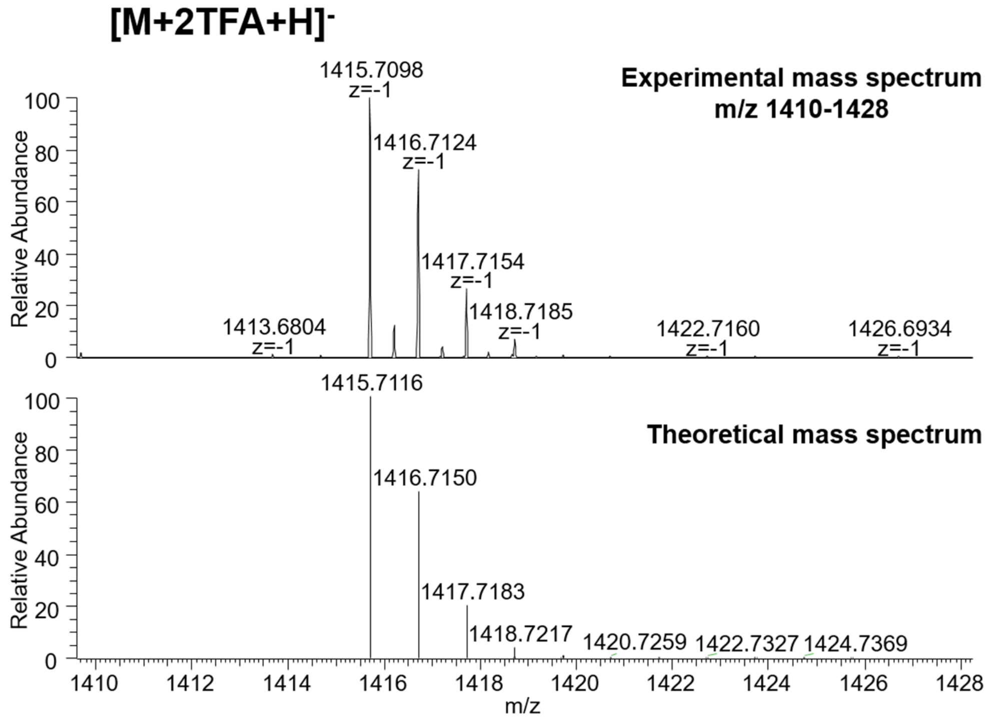

Figure S9: Experimental and theoretical isotopic distribution of the peak corresponding to [M + 2TFA + H]-, $C_{59}H_{97}O_{17}N_{16}F_6$.

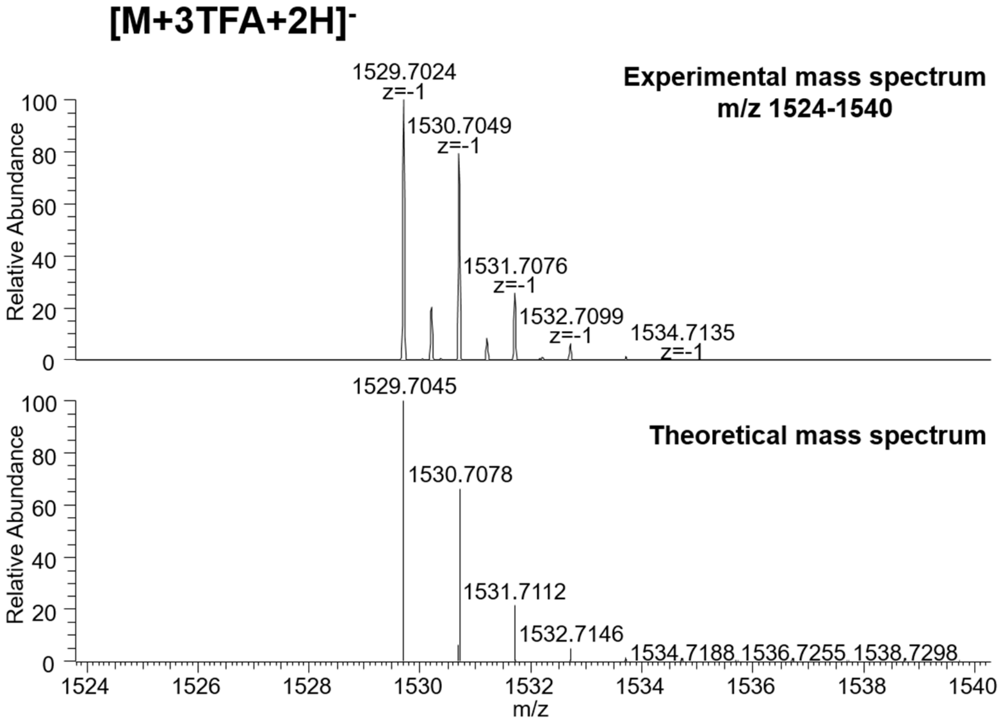

Figure S10: Experimental and theoretical isotopic distribution of the peak corresponding to [M + 3TFA + 2H]$^-$, $C_{61}H_{98}O_{19}N_{16}F_9$.

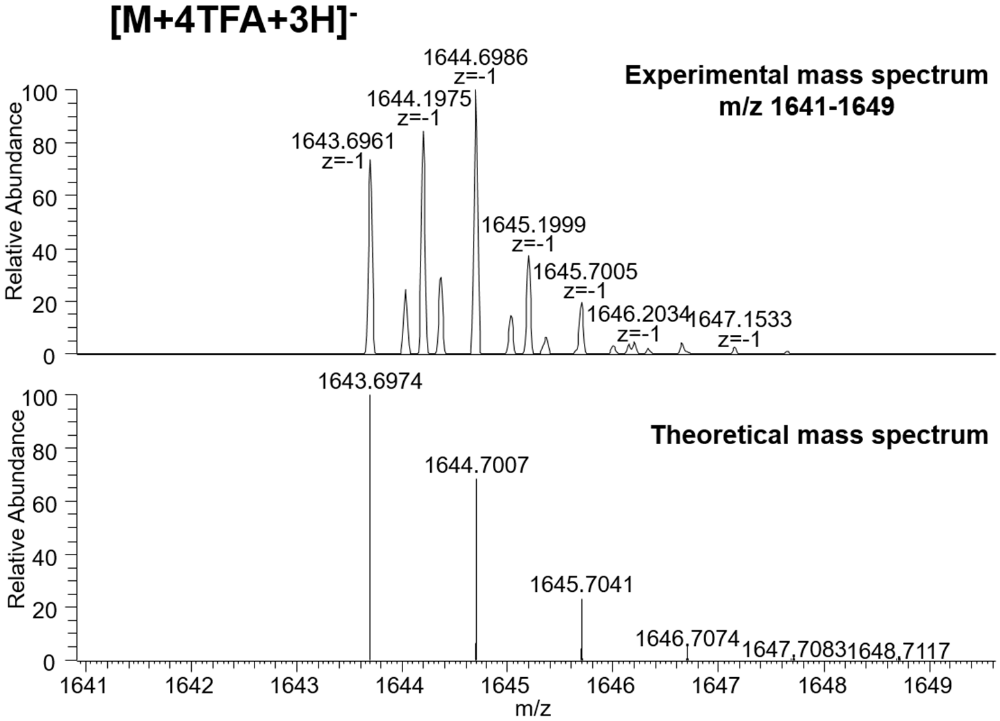

Figure S11: Experimental and theoretical isotopic distribution of the peak corresponding to [M + 4TFA + 3H]$^-$, $C_{63}H_{99}O_{21}N_{16}F_{12}$.

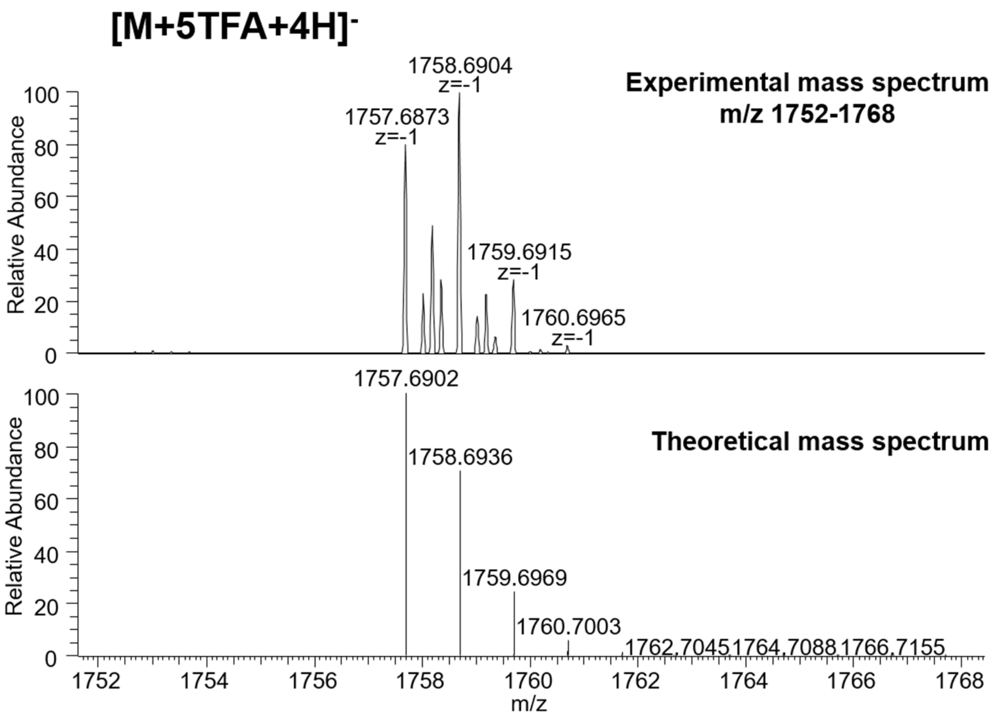

Figure S12: Experimental and theoretical isotopic distribution of the peak corresponding to [M + 5TFA + 4H]-, $C_{65}H_{100}O_{23}N_{16}F_{15}$.

PxB3 after lyophilization with HCl 8 mM (a detailed study of Figure 7C):

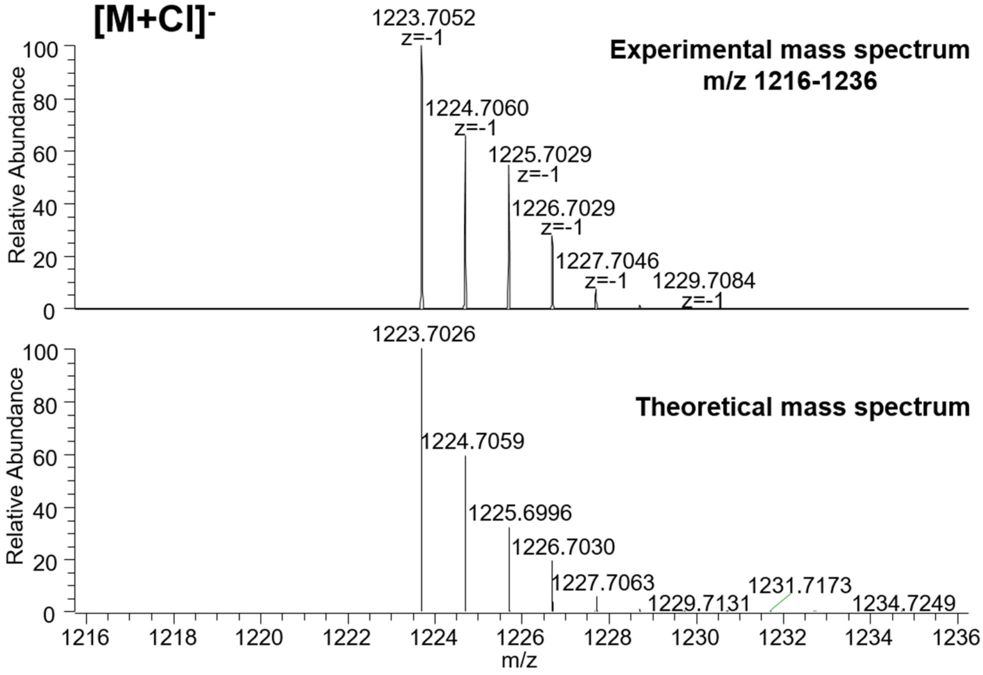

Figure S13: Experimental and theoretical isotopic distribution of the peak corresponding to [M + Cl]-, $C_{55}H_{96}O_{13}N_{16}Cl$.

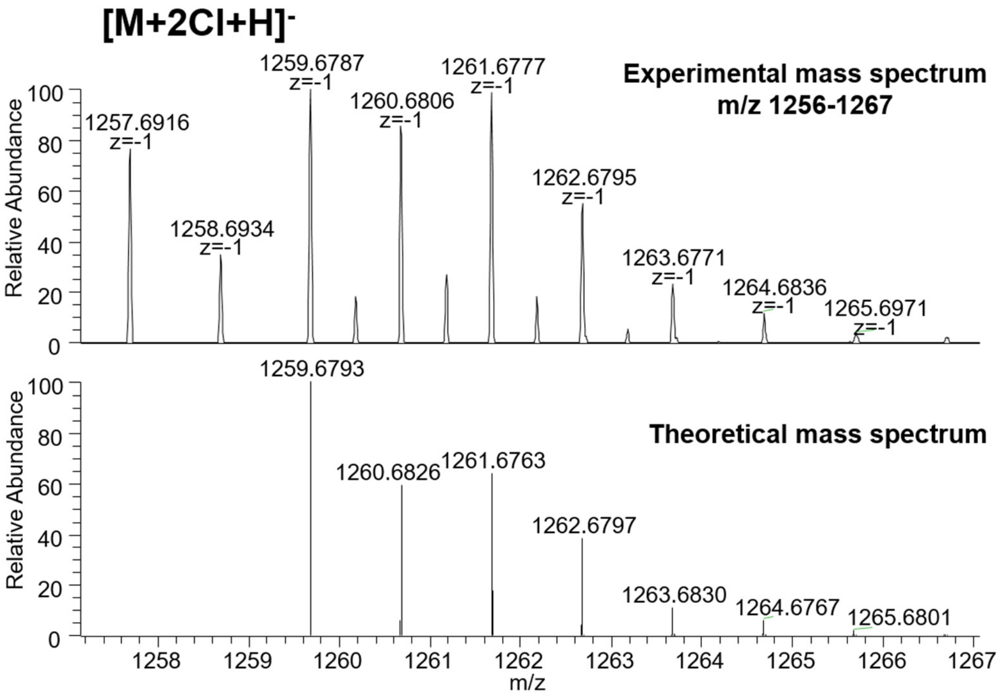

Figure S14: Experimental and theoretical isotopic distribution of the peak corresponding to [M + 2Cl + H]$^-$, $C_{55}H_{97}O_{13}N_{16}Cl_2$.

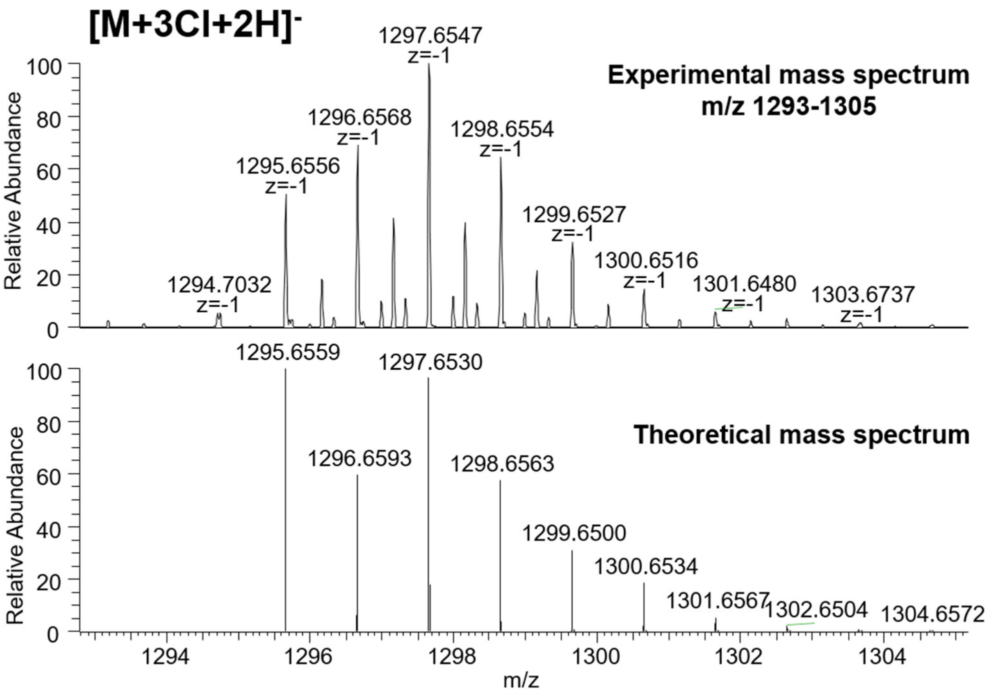

Figure S15: Experimental and theoretical isotopic distribution of the peak corresponding to [M + 3Cl + 2H]$^-$, $C_{55}H_{98}O_{13}N_{16}Cl_3$.

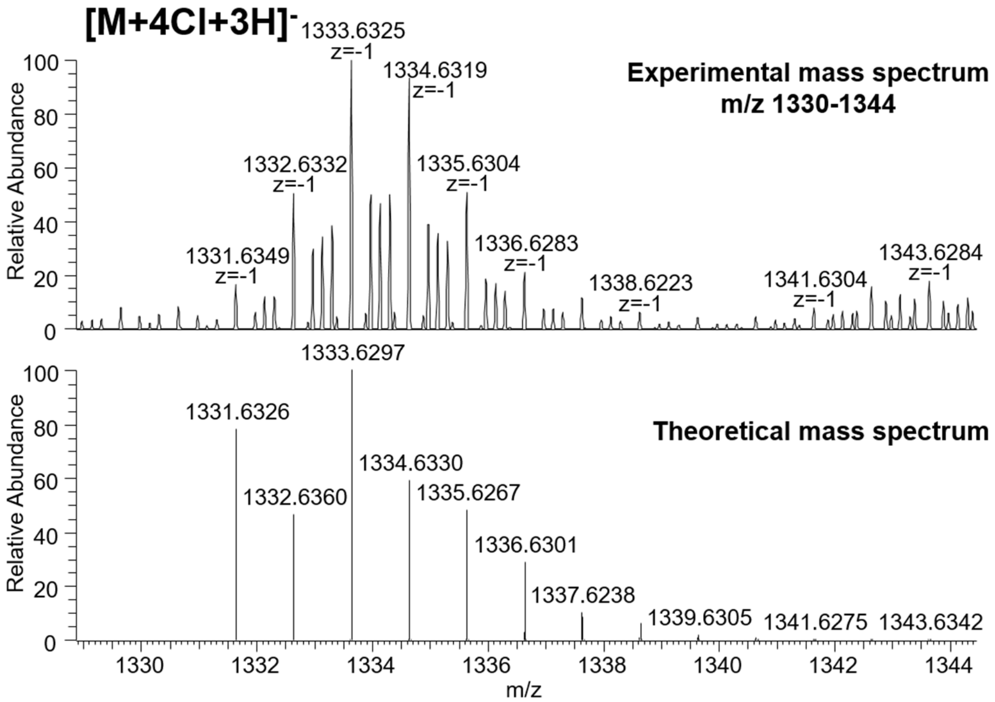

Figure S16: Experimental and theoretical isotopic distribution of the peak corresponding to $[M + 4Cl + 3H]^-$, $C_{55}H_{99}O_{13}N_{16}Cl_4$.

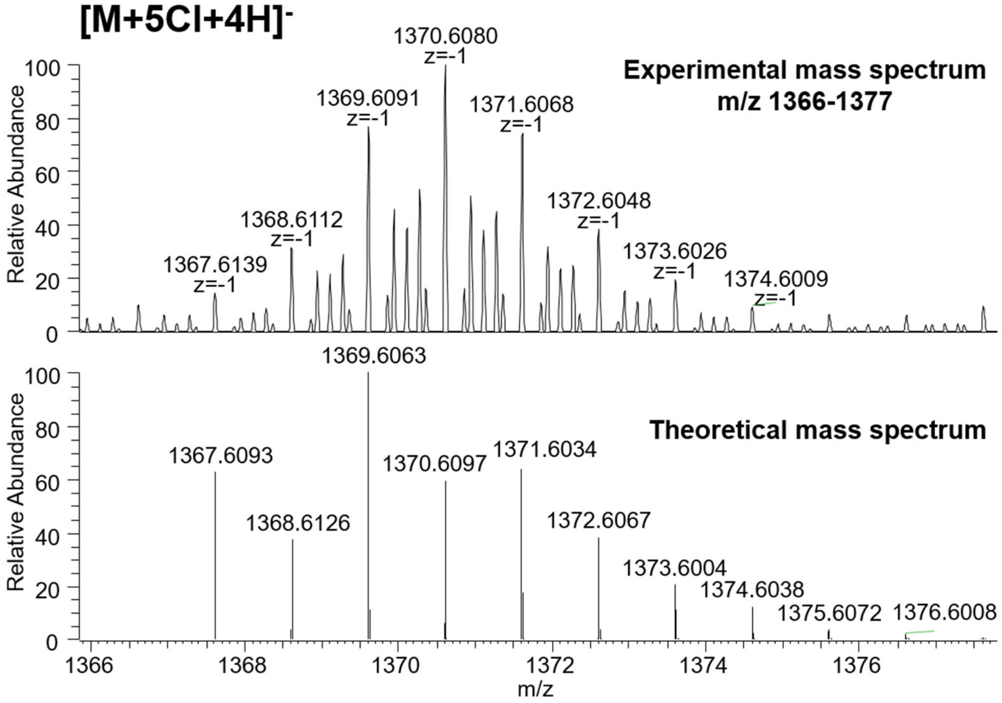

Figure S17: Experimental and theoretical isotopic distribution of the peak corresponding to $[M + 5Cl + 4H]^-$, $C_{55}H_{100}O_{13}N_{16}Cl_5$.

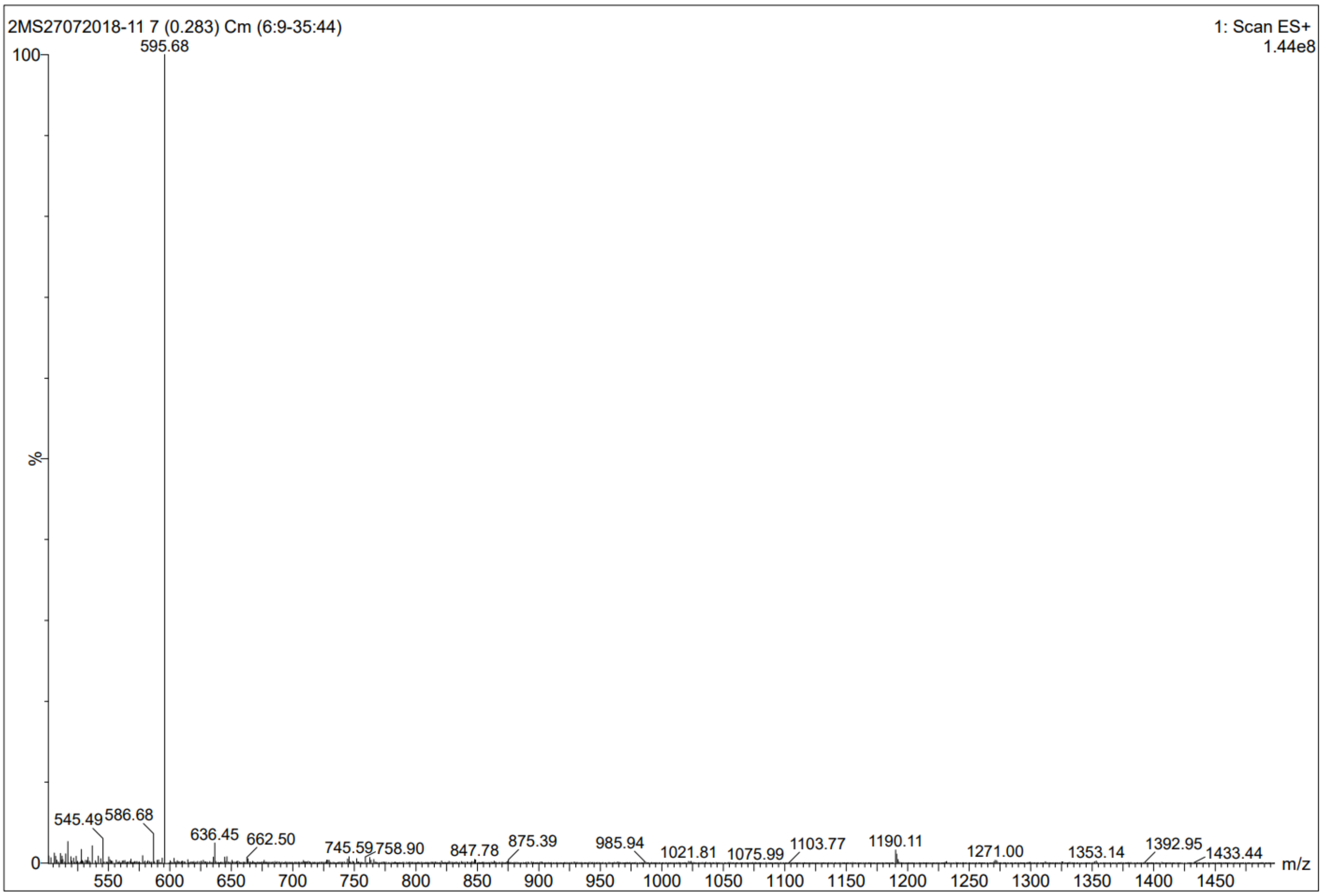

Figure S18: Spectra obtained by ESI-MS in positive mode for polymyxin B_3 peptide.

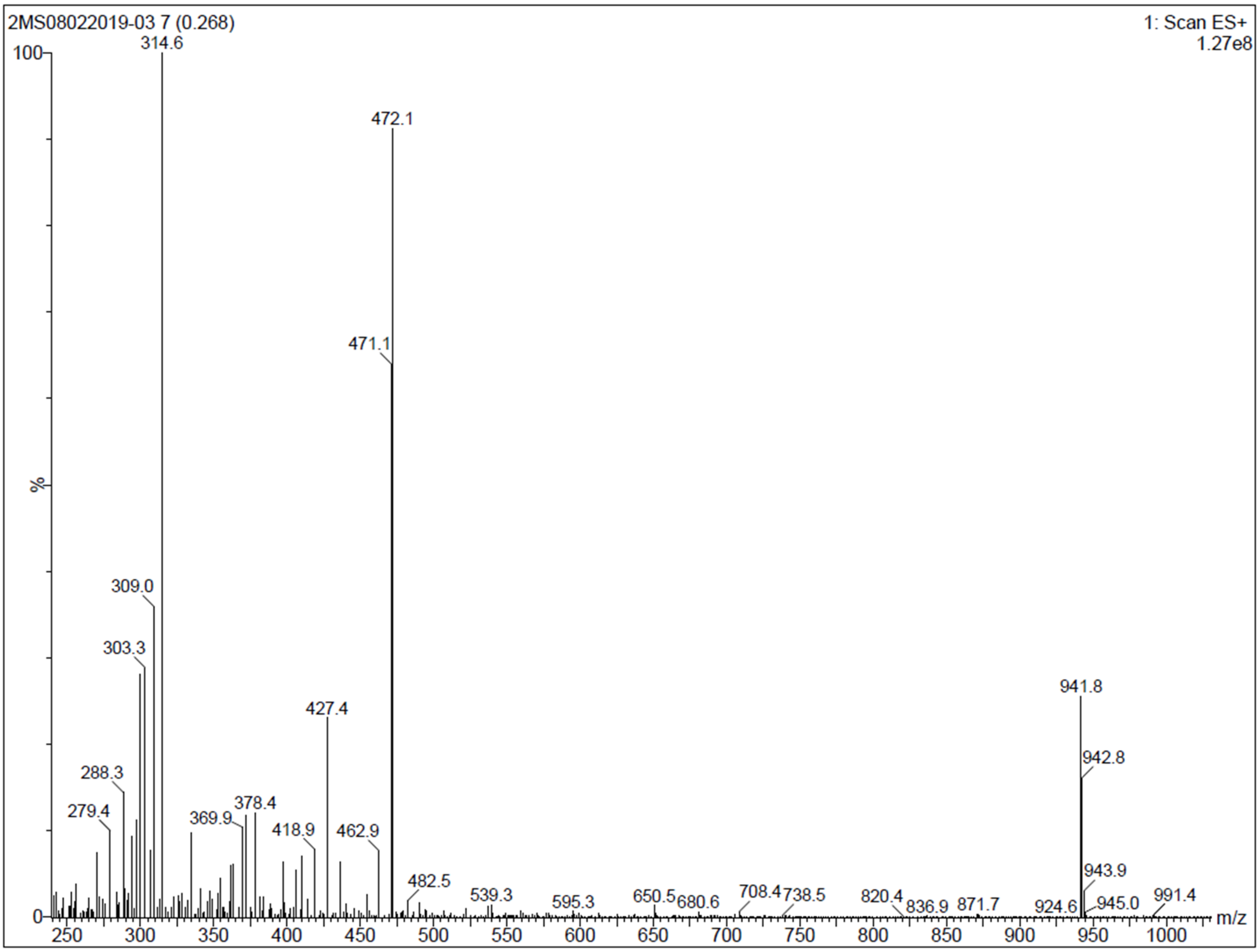

Figure S19: Spectra obtained by ESI-MS in positive mode for RR4 heptapeptide.

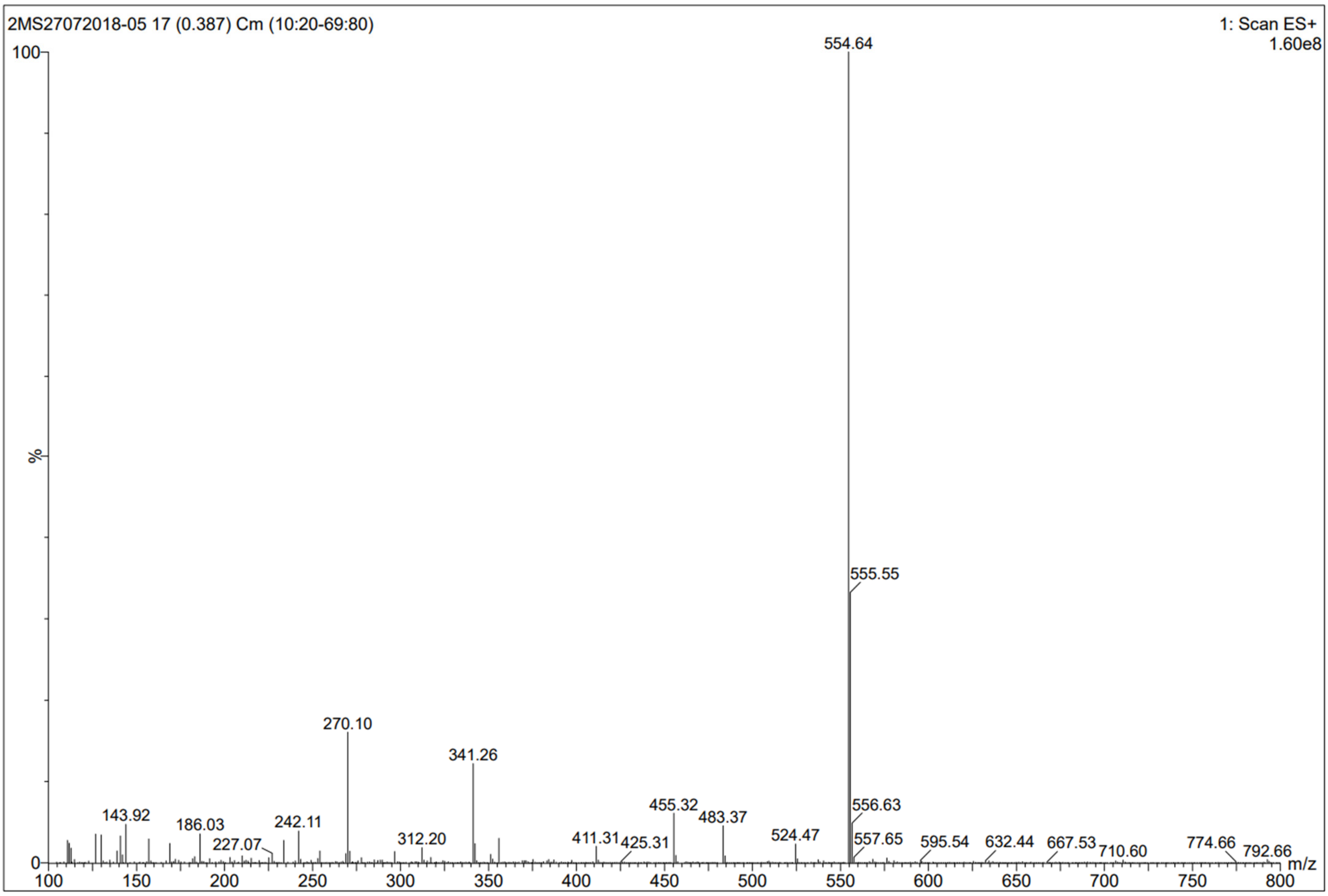

Figure S20: Spectra obtained by ESI-MS in positive mode for the dusquetide peptide.
